# Sialic acid-based probiotic intervention in lactating mothers improves the neonatal gut microbiota and immune responses by regulating sialylated milk oligosaccharide synthesis via the gut–breast axis

**DOI:** 10.1080/19490976.2024.2334967

**Published:** 2024-04-17

**Authors:** Yushuang Wang, Binqi Rui, Xiaolei Ze, Yujia Liu, Da Yu, Yinhui Liu, Zhi Li, Yu Xi, Xixi Ning, Zengjie Lei, Jieli Yuan, Liang Li, Xuguang Zhang, Wenzhe Li, Yanjie Deng, Jingyu Yan, Ming Li

**Affiliations:** a Department of Microecology, College of Basic Medical Science, Dalian Medical University, Dalian, China; b Department of Clinical Laboratory, Central Hospital of Dalian University of Technology, Dalian, China; c Microbiome Research and Application Center, BYHEALTH Institute of Nutrition & Health, Guangzhou, China; d The Third Ward of Obstetrics and Gynecology at Chunliu District, Dalian Women and Children Medical Center (Group), Dalian, China; e Guangdong Provincial Key Laboratory of Infectious Diseases and Molecular Immunopathology, Shantou University Medical College, Shantou, China; f Dalian Institute of Chemical Physics, Chinese Academy of Sciences Key Laboratory of Separation Science for Analytical Chemistry, Dalian, China

**Keywords:** Sialic acid, probiotic, milk oligosaccharide, infant, gut microbiota, immune response, gut–breast axis

## Abstract

Human milk oligosaccharides (HMOs) are vital milk carbohydrates that help promote the microbiota-dependent growth and immunity of infants. Sialic acid (SA) is a crucial component of sialylated milk oligosaccharides (S-MOs); however, the effects of SA supplementation in lactating mothers on S-MO biosynthesis and their breastfed infants are unknown. Probiotic intervention during pregnancy or lactation demonstrates promise for modulating the milk glycobiome. Here, we evaluated whether SA and a probiotic (Pro) mixture could increase S-MO synthesis in lactating mothers and promote the microbiota development of their breastfed neonates. The results showed that SA+Pro intervention modulated the gut microbiota and 6’-SL contents in milk of maternal rats more than the SA intervention, which promoted *Lactobacillus reuteri* colonization in neonates and immune development. Deficient 6’-SL in the maternal rat milk of *St6gal1* knockouts (St6gal1^−/−^) disturbed intestinal microbial structures in their offspring, thereby impeding immune tolerance development. SA+Pro intervention in lactating St6gal1^±^ rats compromised the allergic responses of neonates by promoting 6′-SL synthesis and the neonatal gut microbiota. Our findings from human mammary epithelial cells (MCF-10A) indicated that the GPR41-PI3K-Akt-PPAR pathway helped regulate 6′-SL synthesis in mammary glands after SA+Pro intervention through the gut – breast axis. We further validated our findings using a human-cohort study, confirming that providing SA+Pro to lactating Chinese mothers increased S-MO contents in their breast milk and promoted gut *Bifidobacterium* spp. and *Lactobacillus* spp. colonization in infants, which may help enhance immune responses. Collectively, our findings may help alter the routine supplementation practices of lactating mothers to modulate milk HMOs and promote the development of early-life gut microbiota and immunity.

## Introduction

Human breast milk can provide vital nutrients; bio-active factors, including cytokines, immunoglobulins, human milk oligosaccharides (HMOs); and microorganisms to infants. The effects of these essential ingredients in human milk on infants, such as growth promotion, stimulation of immune development and maturation, and resistance to infections, are closely related to colonization and establishment of early-life gut microbiota.^1^ Thus, manipulating breast milk composition through diet is a promising strategy for the development of healthy intestinal microbiota in newborns, which may confer life-long health benefits.

HMOs are vital milk carbohydrates that help shape the gut-microbial structure and promote the microbiota-dependent growth and protection of infants.^2^ Human milk contains a large amount of sialic acid (SA), 70–83% of which combines with HMOs to form sialylated HMOs (S-HMOs).^3^ S-HMOs are crucial for regulating the intestinal microbial ecosystem and improving the bone and cognitive development of neonates.^4–7^ Lodge et al.^8^ reported that the content of 6′-sialyllactose (6′-SL), one of the core structures of S-HMOs, in the milk of lactating mothers negatively correlated with the occurrence of allergic diseases in neonates, suggested a role for S-HMOs in regulating the neonatal immune responses.

The biosynthesis of mammalian S-HMOs, such as 6′-SL, in the mammary glands mainly involved the conjugation of SA to lactose molecules or basal oligosaccharide structures in reactions catalyzed by sialyltransferases^9^; notably, ST6Gal-1 is a sialyltransferase that controls the modification of α-2,6-linked SA to form sialylated oligosaccharides or glycoconjugates in mammals. Although SA was abundant in human milk during the first month of lactation, its concentration decreased by 70% over the course of 3 months.^10^ Although SA can be synthesized *de novo*, this process may not be sufficient for either HMO biosynthesis by mothers or infant development during lactation.^11^ In addition, our previous study in Chinese mothers showed that, mothers with gestational diabetes mellitus had significantly lower levels of total and specific HMOs (such as S-HMOs) in their colostrum, resulted in delayed colonization of *Lactobacillus* and *Bifidobacterium* spp. in their breast-fed infants.^12^ Therefore, nutritional intervention in lactating mothers might be an unique opportunity to improve aberrant HMOs and early gut microbiome colonization to prevent potential negative health outcomes in children at increased risk due to maternal factors. Although 6′-SL has been added to infant formula in some countries and regions, it is not used on a global scale. In contrast, intervention with edible bird’s nest (a widely used traditional Chinese health food with a high SA content)^13^ during lactation can improve spatial-learning performances in offspring.^14^ However, we lack reports on the effects of dietary SA supplementation on S-HMOs biosynthesis in lactating mothers and neonatal gut microbiota.

Recent data have indicated that inter-organ connectivity influences the quality of a mother’s milk, potentially enabling personalization for her offspring. The maternal gut plays a quintessential role in programming the mammary gland to provide for the multiple requirements of the growing infant. Karcz et al. demonstrated that the composition and diversity of HMOs were closely associated with the maternal diet,^15^ suggestive of functional connections in the gut – breast axis. Probiotics are important modulators of the human gut microecology and nutritional status. The consumption of specific probiotic strains, such as *Bifidobacterium breve M*-16 V,^16^ *Lactobacillus rhamnosus* HN001,^17^ and *Lactobacillus fermentum* CECT5716,^18^ during pregnancy and lactation confers health benefits to mothers and their infants. Seppo et al.^19^ conducted a retrospective study and reported that maternal probiotic supplementation during the late stage of pregnancy altered the HMO composition in the colostrum, suggesting that probiotic intervention during lactation may modulate the HMO contents through the gut – breast axis. However, the effects of probiotic supplementation on HMO patterns during lactation have not been investigated thoroughly. Additionally, the lack of systematic animal and clinical research renders it difficult to clarify the underlying mechanism whereby probiotics regulate HMO synthesis.

Accordingly, we aimed to evaluate the effects of intervention with an SA-probiotic combination (SA+Pro, *B. breve M*-16 V, *L. rhamnosus* HN001, and *L. fermentum* CECT5716) on the diversity and composition of milk oligosaccharides in lactating rat and human mothers, and to further investigate whether changes in maternal milk oligosaccharides can eventually promote the establishment of gut microbiota that beneficial for the immune development of their breastfed neonates. We believe that our results help improve the routine dietary-supplementation practices of lactating mothers to modulate milk HMOs and promote the development of early-life gut microbiota and immunity.

## Results

### SA+Pro intervention modulates sialylated milk oligosaccharides (S-MOs) and the microbiota of maternal rats during lactation

**Figure 1.**
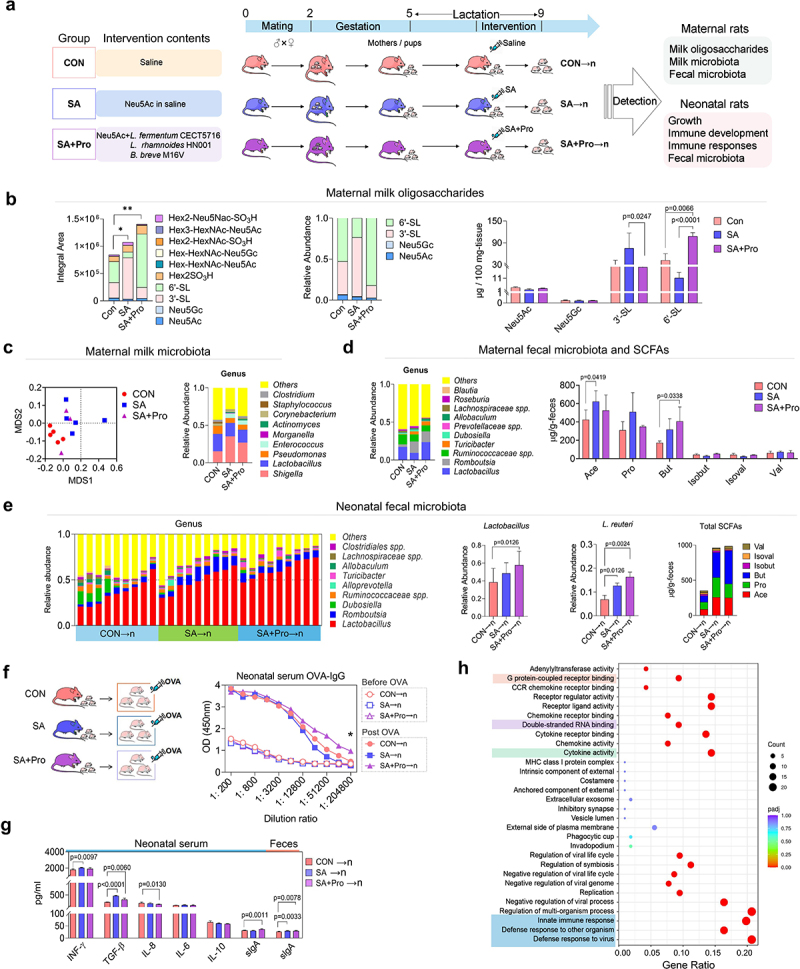
Effects of SA and SA+Pro interventions on maternal and neonatal rats.

Since SA is a key component for the biosynthesis of S-HMOs, and probiotics are important modulators of the maternal gut microecology and may exert functional effects on mammary gland through the gut – breast axis, we therefore firstly evaluated whether intervention of SA combined with probiotics (SA+Pro) could affect milk-oligosaccharides synthesis in lactating Sprague – Dawley rats (Figure 1(a)). Two weeks after delivery, the maternal rats were continually administered intragastric interventions containing saline (control [CON] group), SA, or SA+Pro for 2 weeks, as described in the Materials and Methods section. These interventions did not significantly alter the body weights of the maternal rats but showed a promoting effect on growth of the neonatal rats during late lactation (Figure S1a). Importantly, we found that the rats in the SA and SA+Pro groups had significantly higher total S-MO contents in their milk (Figure 1(b)), which may contributed to the growth promotion of neonates.^2^ The rats in the SA+Pro group exhibited significantly higher 6′-SL milk contents than those in the CON and SA groups.

(a) Schematic representation of the animal study. (b) Effects of SA and SA+Pro interventions on oligosaccharide and free SA levels in rat milk. (c) The β-diversity of milk microbiota represented using NMDS and the relative abundances of bacterial genera in rat milk after intervention with SA and SA+Pro. (d) The relative microbial abundances in the intestinal microbiota and the SCFA compositions in the feces of maternal rats. (e) The abundance of intestinal bacterial genera in neonatal rats. →n was used in figures to differentiate the neonatal groups from the maternal groups; The relative abundances of *Lactobacillus* spp., *L. reuteri*, and total and individual SCFA contents were compared among the different groups of neonatal rats. (f) A schematic representation of the OVA-immunization procedure used with neonatal rats fed by different mothers, as well as the serum levels of OVA-specific IgG (OVA-IgG) in neonatal rats before and after OVA-immunization. (g) The levels of sIgA and cytokines in the serum and feces of neonatal rats. (h) Bubble plots representing the results of GO enrichment analysis based on differentially expressed genes in splenic lymphocytes from *L. reuteri*-treated rats compared with those in untreated lymphocytes. All values are presented as the mean ± SD (*n* ≥ 5).

16S rDNA sequencing analysis revealed shifts in the microbial-clustering trends in the milk of rats that received SA or SA+Pro (Figure 1(c)), which were characterized by a lower abundance of *Pseudomonas* spp. and higher abundances of *Shigella* spp. and *Enterococcus* spp. than that in the CON rats. However, the supplemented probiotic strains were not detected in the milk from maternal rats in the SA+Pro group (Supporting Information, Figure S1b). The gut microbiota of maternal rats in the different intervention groups were also analyzed. SA+Pro intervention resulted in higher abundances of *Lactobacillus* spp., *Romboutsia* spp., and *Turicibacter* spp. than that in the CON group. The levels of total short-chain fatty acids (SCFAs), especially the levels of acetate (Ace) in the SA group and butyrate (But) in the SA+Pro group (Figure 1(d)), in feces of the maternal rats post-intervention were higher than those in the CON group (Supporting Information, Figure S1c). These findings suggest that the gut microbial metabolic profile was altered by these interventions in maternal rats.

### Sa+pro intervention alters the gut microbiota in lactating rats and the immune responses of neonates

Subsequently, we investigated whether changes in S-MOs and the microbiota of the maternal rats caused by intervention affect the intestinal microecology of their offspring. We observed that the abundance of *Lactobacillus* spp. (especially *L. reuteri*) and *Romboutsia* spp. in the neonates fed by rat mothers who received SA+Pro (SA+Pro→n) was significantly higher than that in the control neonates who had fed by CON rat mothers (CON→n) (Figure 1(e)). We also detected *B. breve* in the feces of the neonatal rats (Supporting Information, Figure S1d), indicating its vertical transmission from the maternal gut to the neonatal gut. The levels of fecal SCFAs in the offspring of the intervention groups were significantly higher than those in the CON→n group, especially in terms of Ace and But (Supporting Information, Figure S1e).

To determine whether changes in the gut microbiota of the neonatal rats affected their immune development, we monitored the immune responses of neonatal rats immunized with ovalbumin (OVA; Figure 1(f)). The levels of serum OVA-specific IgG in the SA+Pro→n group of neonates post-OVA immunization were significantly higher than those in the other groups (Figure 1(f)), suggesting that their humoral immune responses were enhanced. Enzyme-linked immunosorbent assay (ELISA) revealed that administering SA or SA+Pro to maternal rats altered the serum-cytokine levels of the neonatal rats. Notably, the serum concentrations of secretory immunoglobulin A (sIgA) were significantly higher in the SA+Pro→n group than that in the SA→n group (Figure 1(g)).

Early-life colonization of beneficial bacteria can promote immune system development and maturation.^20^ To evaluate whether *L. reuteri* was responsible for the enhanced neonatal immune responses, we compared the gene-expression profiles of rat splenic lymphocytes stimulated with or without *L. reuteri* metabolites, using RNA sequencing (RNA-Seq). Gene Ontology (GO)-based enrichment analysis indicated that treatment with the *L. reuteri* metabolites induced genes involved in the innate immune responses, cytokine activity, and multiple signal pathways (such as those related to G protein-coupled receptors [GPCRs] and double-stranded RNA-binding) in splenic lymphocytes (Figure 1(h)), suggesting the potential of *L. reuteri* to promote immune responses in neonatal rats.

### Sa+pro intervention in lactating St6gal1^±^ rats compromise the allergic responses of neonates by regulating 6′-SL synthesis and the neonatal gut microbiota

**Figure 2.**
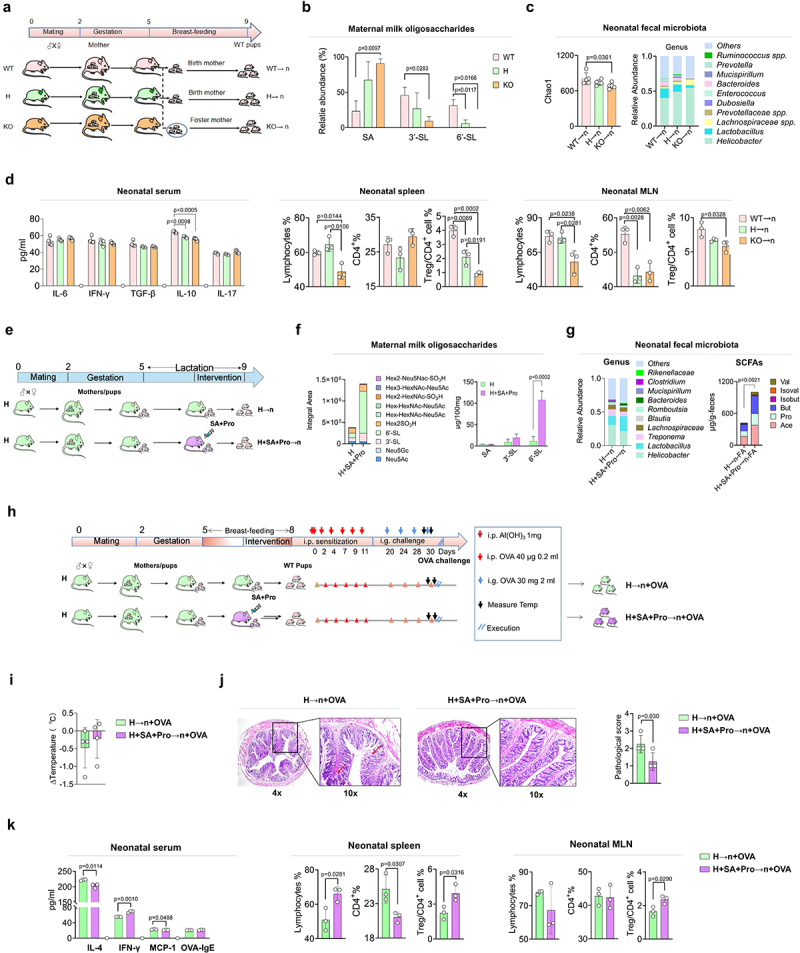
SA+Pro intervention in lactating St6gal1^±^ (H) rats compromised allergic responses in neonates by regulating 6′-SL synthesis and the neonatal gut microbiota.

To determine the effects of 6′-SL on the intestinal microbiota and immune development in offspring, we established St6gal1^−/−^ knockout (KO) and St6gal1^±^ (heterozygous, H) rat models (Supporting Information, Figure S2); female rats were used for the cross-feeding experiments (Figure 2(a)). The St6gal1 KO rats had significantly lower S-MO levels in their milk than did the wild-type (WT) rats, and the 6′-SL contents were substantially diminished or abolished in the milk of the maternal H or KO rats, respectively (Figure 2(b)).

(a) A schematic diagram depicting the cross-feeding procedure used with WT neonates fed by WT, H, and KO maternal rats. (b) Comparison of the major milk oligosaccharides in different rat mothers. (c) Changes in the intestinal microbiota of neonates fed by different maternal rats. (d) The serum levels of cytokines in neonatal rats and changes in lymphocyte subsets in the spleen and neonatal MLNs. (e) A schematic diagram indicating that neonates fed by H maternal rats or H maternal rats received SA+Pro intervention. (f) Changes in the major milk oligosaccharides of the H maternal rats, with or without SA+Pro intervention. (g) The abundance of intestinal bacterial genera and the total and individual SCFA contents in neonatal rats. (h) A schematic diagram representing the establishment of a model of OVA-induced allergic responses in neonates fed by H maternal rats, with or without SA+Pro intervention. (i) Differences in the anal temperatures of neonatal rats following OVA challenge. (j) Hematoxylin and eosin staining and histopathological scores of the intestines in the offspring. (k) Changes in serum anaphylaxis-related cytokine levels, expressed as a percentage of the lymphocyte subpopulations in the spleen and MLNs of offspring. All values are presented as the mean ± SD (*n* ≥ 3).

Deficient 6′-SL in rat milk resulted in a lower intestinal microbial diversity (represented by Chao1 index) in offspring rats compared with that in rats fed by WT mothers (Figure 2(c)). The deficiency also altered the microbial structure by reducing the abundance of *Lactobacillus* spp. (mainly *L. reuteri*, Supporting Information) and increasing the abundance of other bacteria, such as *Helicobacter* spp. and *Lachnospiraceae* spp. Further analysis of the immune parameters of neonatal rats revealed that the serum levels of interleukin (IL)-10 in the H→n and KO→n groups were significantly lower than that in the WT→n group (Figure 2(d)). The proportion of total lymphocytes in the spleen and mesenteric lymph nodes (MLNs) of the KO→n group was significantly lower than those of the other groups. Notably, the proportions of regulatory T (Treg)/CD4+ cells in the spleen and MLNs were significantly lower in the KO→n group than that in the WT→n group. Intestinal Treg cells play important roles in regulating the early-life establishment of immune tolerance. These results suggest that 6′-SL deficiency-induced alterations in the neonatal gut microbiota may have impeded the development of immune tolerance in offspring rats.

To explore the effect of maternal SA+Pro intervention on S-MO synthesis in St6gal1^±^ (H) rats and the gut microbiota of their neonates, we administered SA+Pro to H rats mothers during lactation for 2 weeks (Figure 2(e)). SA+Pro supplementation to St6gal1^±^ maternal rats (H+SA+Pro group) significantly enhanced the total S-MO and 6′-SL contents in their milk compared with those of the non-treated H rats (Figure 2(f)). Analysis of the intestinal microbiota in the gut of the neonatal rats indicated that the abundance of *Lactobacillus* spp., mainly *L. reuteri* (Supporting Information, Figure S3), was higher in the H+SA+Pro→n group than that in the H→n group, whereas the abundance of *Helicobacter* spp. was lower, resulting in elevated fecal SCFA levels post-intervention (Figure 2(g)).

To further evaluate the effects of maternal SA+Pro intervention on the development of immune tolerance in offspring, we induced an allergic response (OVA challenge) in offspring rats fed by H mothers treated with or without SA+Pro during lactation (Figure 2(h)). After the OVA challenge, the neonates fed by H rats exhibited severe allergic responses, such as a decreased body temperature (Figure 2(i)), severe intestinal pathological changes (Figure 2(j)), and elevated circulating IL-4 and monocyte chemoattractant protein-1 levels compared with those in the CON rats (WT→n, Figure 2(k)). In contrast, SA+Pro intervention compromised these changes toward levels comparable with those of the CON group. Furthermore, SA+Pro intervention in lactating H rats increased the proportion of splenic and MLN Treg/CD4+ cells in neonates (Figure 2(k)).

### Gpr41-PI3K-Akt-PPAR pathway is involved in regulating 6′-SL synthesis in the mammary gland after SA+Pro intervention

**Figure 3.**
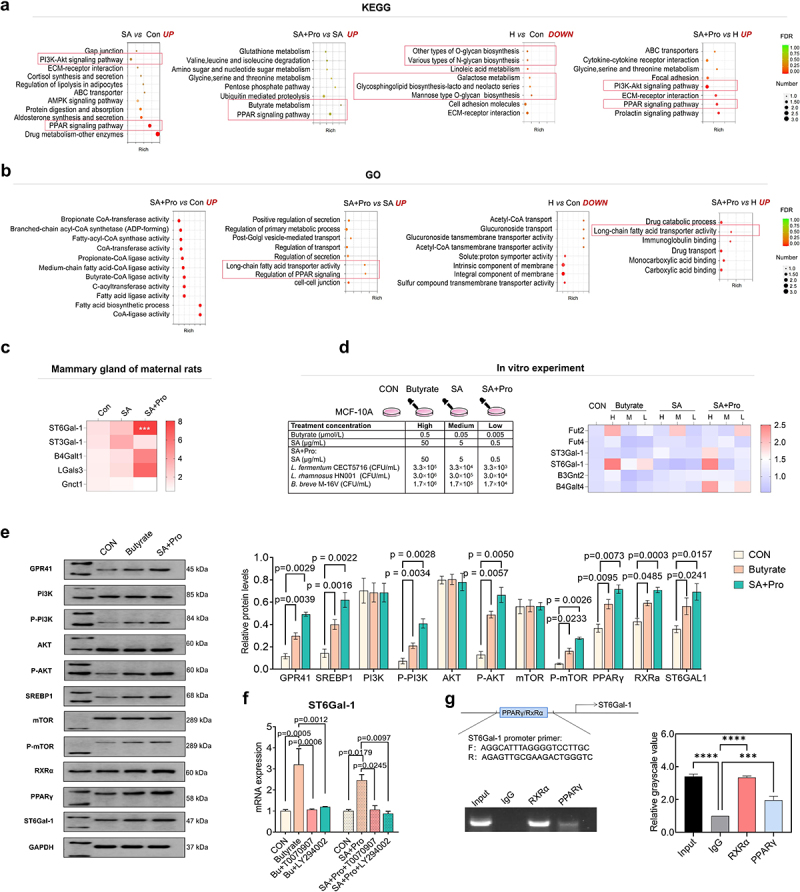
The Gpr41-PI3K-Akt-PPAR pathway was involved in regulating 6′-SL biosynthesis in mammary glands by SA+Pro.

To further explore the mechanism whereby SA+Pro treatment promoted 6′-SL synthesis, we identified the differentially expressed genes in rat mammary glands using RNA-Seq analysis. Kyoto Encyclopedia of Genes and Genomes (KEGG) analysis revealed that genes involved in PI3K-Akt and PPAR signaling pathways were expressed at substantially higher levels in the breast tissues of maternal rats in the SA group, compared with those in the CON group (Figure 3(a)). Compared with SA intervention alone, SA+Pro intervention significantly promoted the transcription of genes involved in butyrate (But) metabolism and the PPAR signal pathway. Genes involved in synthesizing various glycans were expressed at lower levels in the mammary glands ^21–26^ of the H (St6gal1^±^) group than in those of the CON group. However, after SA+Pro supplementation, genes involved in PI3K-Akt and PPAR signaling pathways were expressed at significantly higher levels in the H group. Furthermore, GO enrichment analysis suggested that SA+Pro intervention upregulated genes involved in medium and long-chain fatty acids synthesis and transportation (Figure 3(b)). We further evaluated the expression levels of several glycosyltransferases in rat mammary glands (which are responsible for milk oligosaccharide synthesis) and observed that SA+Pro intervention had significantly upregulated *St6gal1* expression (Figure 3(c)). These results suggest that PI3K-Akt and PPAR signaling pathways, as well as long-chain fatty acids, play vital roles in regulating *St6gal1* expression and 6′-SL biosynthesis in rat mammary glands.

(a) Bubble plots representing KEGG enrichment analysis of differentially expressed genes related to signaling pathways in the mammary glands of different groups. The CON group indicated the mammary glands of the WT maternal mice, the H groups indicated the mammary glands of the H maternal mice, and the SA and SA+Pro groups indicated the mammary glands of the maternal mice that received SA and SA+Pro intervention during lactation, respectively. (b) Bubble plots represent GO enrichment analysis of differentially expressed genes in the mammary glands of different groups. (c) Expression of glycosyltransferases in the mammary glands of rats in different groups. (d) Effects of different treatments on the expression of glycosyltransferases in human mammary epithelial cells (MCF-10A). (e) The expression and phosphorylation levels of proteins involved in the PI3K-AKT-mTOR and PPARγ pathways were determined using western blot analysis. GAPDH expression was detected as an internal control. The protein expression levels were quantified using densitometry. (f) Effects of PPARγ and PI3K-AKT inhibitors on ST6Gal-1 expression in MCF-10A cells stimulated with But. (g) ChIP assay results depicting the interaction between the RXRα–PPARγ complex and the promoter region of *St6gal1*. All values are presented as the mean ± SD (*n* ≥ 3), ***p < .001.

As SA+Pro intervention considerably enhanced But production in the maternal gut, we hypothesized that the uptake of circulating But into rat mammary glands may have helped promote *St6gal1* expression by activating the PI3K-Akt and PPAR signaling pathways. To validate this hypothesis, we stimulated human mammary epithelial MCF-10A cells directly with SA, SA+Pro, and But to evaluate their effects. The results suggested that SA+Pro and But promoted the expression of *St6gal1* and other glycosyltransferase genes such as *Fut2* and *B4galt4* (Figure 3(d)).

Western blot analysis of MCF-10A cells treated with or without treatment of SA+Pro and But revealed that the expression or phosphorylation levels of GPR41, PI3K, AKT, mTOR, SREBP1, RXRα, PPARγ, and ST6Gal-1 were increased by SA+Pro and But stimulation (Figure 3(e)). ST6Gal-1 expression was no longer promoted by either SA+Pro or But treatment after treatment with PPARγ and PI3K-Akt inhibitors (T0070907 and LY294002, respectively; Figure 3(f)). Chromatin immunoprecipitation (ChIP) assays confirmed that the RxRα–PPARγ complex was bound to the ST6Gal-1 promoter region (Figure 3(g)).

Collectively, these results indicate that SA+Pro promoted circulating But levels, which stimulated the GPR41-PI3K-AKT-mTOR pathway to promote phosphorylated mTOR (p-mTOR) production in mammary glands. p-mTOR upregulation stimulated SREBP1 nuclear ectopia, as well as long-chain fatty acids biosynthesis and transportation, which in turn affected the expression of PPARγ (a fatty acid receptor). Finally, RXRα–PPARγ complex formation promoted ST6Gal-1 expression by binding to its promoter region (Figure S4).

### Effects of SA+Pro supplementation on HMOs and the gut microbiota of Chinese mothers during lactation

The results of our animal experiments suggested that SA+Pro intervention contributed to the elevation of S-MOs in rat mothers, which could further promote beneficial-microbe colonization and immune responses in neonates. Therefore, we conducted a randomized, controlled, double-blind supplementary trial of mother – child pairs in North China (Dalian, Liaoning, Figure S5). After a 4-week intervention with SA+Pro in the mothers, the average weight of infants in the neonatal SA+Pro group (N2) was not statistically significantly higher to placebo group (N1) (Table 1). A trend toward elevated saliva sIgA levels was observed in N2 group infants, suggesting that immune responses were promoted in these infants.

**Table 1.** Characteristics of the study participants.

Variable*	Placebo group (M1) (*n* = 34)	Intervention group (M2) (*n* = 32)	*p*-value
Maternal characteristics			
Lactation day (number of milk samples/number of feces samples)			
Day 8	32/29	31/25	
Day 22	32/28	31/27	
Day 36	29/26	30/28	
Age (years)	32.18 ± 4.536	31.72 ± 3.718	.6566
Height (cm)	162.7 ± 5.101	162.4 ± 7.396	.8636
Puerperal weight (kg) Puerperal BMI (kg/m^2^)	65.39 ± 6.943 24.73 ± 2.689	64.78 ± 12.03 23.58 ± 3.385	.8011 .1385
Gestational age (weeks)	39.08 ± 1.047	39.58 ± 0.938	.0453
Pregnancy complication (n, %)	11 (32.4)	14 (43.8)	
Number of pregnancies (n, %)			
Primiparous	27 (79.4)	24 (75)	
Multiparous	7 (20.6)	8 (25)	
Gene (n, %)			
Secretor gene + (SE+)	26 (76.5)	25 (78.1)	
Secretor gene – (SE−)	8 (23.5)	7 (21.9)	
Prenatal use of Abx (n, %)	7 (20.6)	6 (18.8)	
Postpartum use of Abx (n, %)	8 (23.5)	6 (18.8)	
Infant characteristic			
Lactation day (number of feces samples/number of saliva samples)			
Day 8	33/9	31/5	
Day 22	33/−	31/−	
Day 36	30/5	32/7	
Mode of delivery (n, %)			
Vaginal birth	28 (85.3)	25 (78.1)	
Cesarean section	5 (14.7)	7 (21.9)	
Sex (n, %)			
Male	17 (48.6)	17 (53.1)	
Female	18 (51.4)	15 (46.9)	
Apgar score	10	10	
Birth weight (g)	3280 ± 350.0	3386 ± 445.1	.2807
Birth length (cm)	50.51 ± 2.106	50.09 ± 2.022	.4083
Weight on day 42 (g)	4160 ± 371.7	4343 ± 520.7	.1220
Diseases during the intervention period (n, %)	7 (20.6)	5 (15.6)	
Skin rash (days)	2 (2–3)	0	
Eczema (days)	3 (5–6)	3 (4–5)	
Diarrhea (times)	2 (5–6)	2 (5–6)	
Breastfeeding (n, %)			
Breastfed	25 (73.5)	21 (65.7)	
Not exclusively breastfed	9 (26.5)	11 (34.4)	
Salivary sIgA			
Day 8	63.69 ± 36.10	63.86 ± 44.97	.9941
Day 36	55.03 ± 26.78	89.36 ± 43.28	.1496

*The data related to the characteristics of participants were collected at randomization unless otherwise noted. Values are presented as the mean ± standard deviation unless otherwise noted.

**Figure 4.**
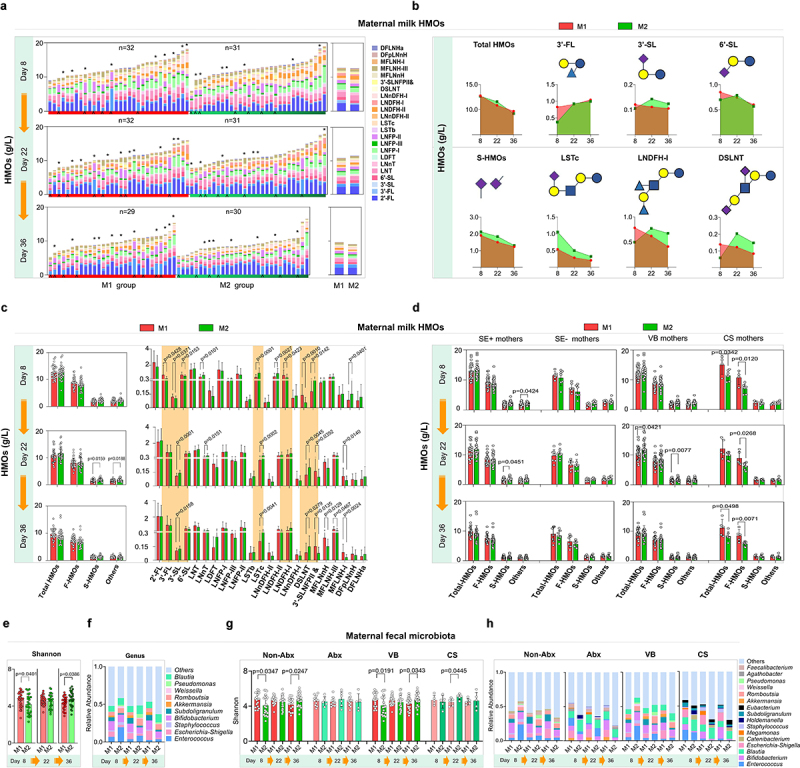
Dynamics of the major HMO fractions and gut microbiota in participants.

After the SA+Pro intervention, the HMO compositions in all milk samples obtained from Chinese mothers were investigated using mass spectrometry. The average total-HMO levels in the mothers of each group were comparable on day 8 and decreased significantly during lactation (Figure 4(a)). We performed a categorical analysis of HMOs and observed that on day 22, the S-HMO levels were significantly higher in the intervention group (the M2 group) than in the placebo group (the M1 group), particularly in terms of 3′-SL, 6′-SL, sialyllacto-*N*-neotetraose c (LSTc), and disialyllacto-N-tetraose (DSLNT), the levels of which were initially lower than those in M1 group but subsequently increased sharply. No significant differences in the total fucosylated HMOs (F-HMOs) levels were observed between both groups. We also observed that SA+Pro intervention promoted the level of 3′-FL by day 36 (Figure 4(b, c)). The 23 major HMO structures identified in the breast milk of Chinese mothers were further assessed (Table 2). The levels of 3′-FL, 3′-SL, 6′-SL, LNDFH, and DSLNT of mothers in the M2 group were initially much lower than those in the M1 group initially (on day 8), whereas the levels of LSTc, LNDFH-II and 3′-SLNFPII & 6′-SLNFPVI were higher (Figure 4(c)). However, after 2 weeks of intervention with SA+Pro, the levels of 2′-FL, 3′-FL, 3′-SL, 6′-SL, and DSLNT increased to varying degrees (especially 3′-SL and DSLNT). We further categorized factors that might influence HMO levels, such as mothers’ secretory type (SE+ or SE−) or delivery mode (vaginal birth [VB] or cesarean section [CB]). SA+Pro intervention increased the S-HMOs levels in both the SE+ and VB mothers (Figure 4(d)).

(a) The contents of the major HMO fractions in the breast milk of each participant (left) and the average HMO levels (right) in each intervention group during the intervention period. *Mothers who used Abx before giving birth or postpartum. ^Non-secretor mothers. (b) Trends of the major HMOs in the milk of participants who received different interventions. (c) Comparison of the contents of all HMOs, F-HMOs, S-HMOs, and other HMOs (neither fucosylated nor sialylated) in the major HMOs fractions in all milk samples from the M1 and M2 groups at three time points. (d) The contents of all HMOs, F-HMOs, S-HMOs, and other HMOs (neither fucosylated nor sialylated) in the milk of secretory and non-secretory VB and CS mothers in the M1 or M2 groups. (e, f) The bacterial α-diversity (indicated using the Shannon index) and the relative abundances of intestinal microbial genera of mothers in each group before and after the intervention. (g, h) The α-diversity of the intestinal microbiota of mothers (indicated using the Shannon index) and the relative abundances of bacterial genera in the gut of secretory and non-secretory VB and CS mothers in the M1 and M2 groups. All values are presented as the mean ± SD. The study size is indicated in the panels. Multiple comparisons were performed by using one-way ANOVA. All values are presented as the mean ± SD (*n* ≥ 20).

**Table 2.** Structures of the major HMOs.

Abbreviation	Trivial name	Structure
2′-FL	2′-Fucosyllactose	Fucα1-2 Galβ1-4Glc
3′-FL	3′-Fucosyllactose	Galβ1–4(Fucα1–3) Glc
3′-SL	3′-Sialyllactose	NANAα2-3 Galβ1-4Glc
6′-SL	6′-Sialyllactose	NANAα2-6 Galβ1-4Glc
LNT	Lacto-*N*-tetraose	Galβ1-3GlcNAcβ1–3 Galβ1-4Glc
LNnT	Lacto-*N-neo*-tetraose	Galβ1-4GlcNAcβ1–3 Galβ1-4Glc
LDFT	Difucosyllactose	Fucα1-2 Galβ1–4(Fucα1–3) Glc
LNFP-I	Lacto-*N*-fucopentaose I	Fucα1-2 Galβ1-3GlcNAcβ1-3 Galβ1-4Glc
LNFP-II	Lacto-*N*-fucopentaose II	Galβ1–3(Fucα1–4) GlcNAcβ1–3 Galβ1-4Glc
LNFP-III	Lacto-*N*-fucopentaose III	Galβ1–4(Fucα1–3) GlcNAcβ1–3 Galβ1-4Glc
LSTb	Sialyllacto-*N*-tetraose b	NANAα2–6(Galβ1–3) GlcNAcβ1-3 Galβ1-4Glc
LSTc	Sialyllacto-*N*-tetraose c	NANAα2-6 Galβ1-4GlcNAcβ1-3 Galβ1-4Glc
LNDFH-I	Lacto-*N*-difucohexaose I	Fucα1-2 Galβ1–3(Fucα1–4) GlcNAcβ1-3 Galβ1-4Glc
LNDFH-II	Lacto-N-difucohexaose II	Galβ1–3(Fucα1–4) GlcNAcβ1-3 Galβ1–4(Fuc1–3) Glc
LNnDFH-I	Lacto-*N-neo*-difucohexaose I	(Fucα1–2) Galβ1–4(Fucα1–3) GlcNAcβ1-3 Galβ1–4 Glc
LNnDFH-II	Lacto-*N-neo*-difucohexaose II	Galβ1–3(Fucα1–4) GlcNAcβ1-3 Galβ1–4(Fucα1–3) Glc
DSLNT	Disialyllacto-*N*-tetraose	NANAα2-6 Galβ1–3(NANAα2–6) GlcNAcβ1-3 Galβ1-4Glc
3′SLNFPII &	3′-Sialyl-LNFP II & 6′-Sialyl-LNFP VI	NANAα2-3 Galβ1–3(Fucα1–4) GlcNAcβ1–3 Galβ1-4Glc & NANAα2-6 Galβ1–4 GlcNAcβ1-3 Galβ1–4(Fucα1–3) Glc
MFLNnH	Mono-fucosyl-LNnH	Galβ1–4(Fucα1–3) GlcNAcβ1–3 (Galβ1-4GlcNAcβ1–6) Galβ1-4Glc
MFLNH-I	Mono-fucosyl-LNH-I	Fucα1-2 Galβ1-3GlcNAcβ1–3(Galβ1-4GlcNAcβ1–6) Galβ1-4Glc
MFLNH-III	Mono-fucosyl-LNnH-III	Galβ1-3GlcNAcβ1–3(Galβ1–4[Fucα1–3] GlcNAcβ1–6) Galβ1-4Glc
DFpLNnH	Difucosyl-*para*-LNnH	Galβ1–4(Fucα1–3) GlcNAcβ1-3 Galβ1–4 (Fucα1–3) GlcNAcβ1-3 Galβ1-4Glc
DFLNHa	Difucosyl-LNHa	Fucα1-2 Galβ1-3GlcNAcβ1–3(Galβ1–4[Fucα1–3] GlcNAcβ1–6) Galβ1-4Glc

Fuc: fucose; Gal: galactose; Glc: glucose; GlcNAc: N-acetylglucosamine; NANA: *N*-acetylneuramic acid (sialic acid).

The gut microbiomes of the mothers were also assessed. SA+Pro intervention progressively increased the α diversity of the gut microbiota (represented by Shannon index) in the M2 group of mothers after a 2–4-week SA+Pro intervention (Figure 4(e)). The abundance of *Enterococcus* and *Escherichia-Shigella* in their guts decreased significantly (Figure 4(f)). Further analysis revealed that SA+Pro effectively promoted the α diversity of gut microbiota in mothers who did not receive antibiotics (the non-Abx M2 group), but not in mothers who received antibiotics (the Abx group, Figure 4(g)). The intervention helped reduce the abundance of *Enterococcus* spp. in non-Abx mothers and *Blautia* spp. in mothers who received Abx. SA+Pro intervention promoted the diversity of the gut microbiota in mothers regardless of their mode of birth (Figure 4(g)) and reduced the abundance of *Enterococcus* and *Holdelanella* spp. in VB mothers (Figure 4(h)).

### SA+Pro intervention in lactating Chinese mothers promotes bifidobacterium colonization in the gut of infants

Considering the alterations of milk HMOs in mothers, we further investigated the gut microbiota and health status of infants fed by mothers in different intervention groups. Counterintuitively, we observed that fewer skin rashes were present in the N2 group of infants (whose mothers received SA+Pro intervention) than in the N1 group (whose mothers received placebo intervention) and that the eczema duration of infants in the N2 group was also reduced (Table 1). Furthermore, although we did not collect a large number of samples and received only a few qualified infant saliva samples, the results suggested that the saliva sIgA levels of the SA+Pro group were significantly higher than those of the placebo group.

**Figure 5.**
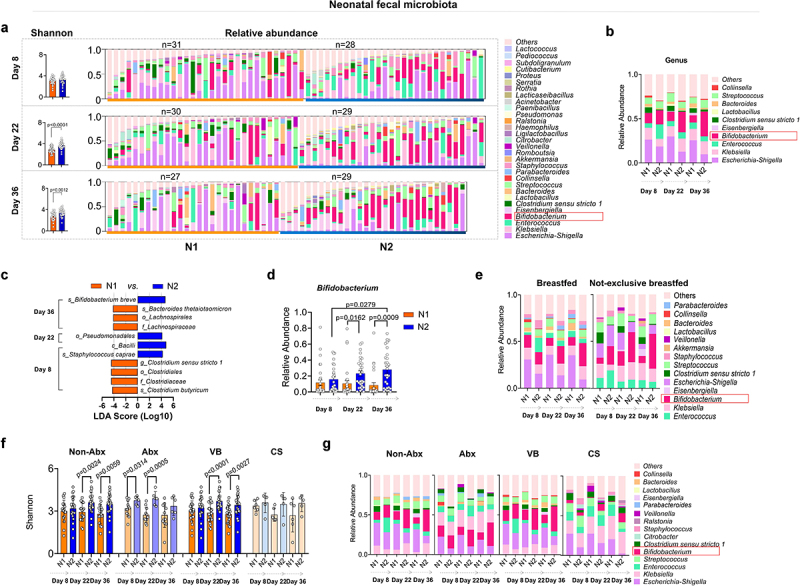
Changes in the intestinal microbiota of infants fed by different mothers.

We further monitored the gut microbiota of the infants. Initially, on day 8, no difference was observed in the bacterial diversity (Shannon index) between both groups of infants (Figure 5(a)). However, by day 22 and day 36, the Shannon index of the N2 group (fed by mothers in the M2 group who received SA+Pro intervention) was significantly higher than that of the N1 group (fed by mothers in the M1 group who received a placebo). The abundances of *Enterococcus* and *Escherichia-Shigella* in the N2 infants were evidently lower than those in N1 infants. Furthermore, the abundance of *Bifidobacterium* in the gut of these infants was significantly increased (Figure 5(b)). Linear discriminant analysis effect size (LEfSe) analysis indicated that the abundance of *B. breve* increased significantly in the N2 group by day 36 (Figure 5(c)) and that the abundance of *Bifidobacterium* in the N2 group displayed a significant upward trend (Figure 5(d)). Notably, SA+Pro intervention in mothers had a considerable impact on infants that were not exclusively breastfed (Figure 5(e)). In addition, we found that intervention of SA+Pro to mothers during lactation who had not used antibiotics or had vaginal delivery was more effective in upregulating the gut bacterial diversity in their breast-fed infants (Figure 5(f)). However, for mothers who had taken antibiotics or had undergone cesarean section, SA+Pro intervention did not significantly increase the gut microbiota diversity of their newborns. This may be due to a small number of enrolled samples and a higher diversity in the N2 group of infants initially at day 8. Nevertheless, a promoting pattern of *Bifidobacterium* spp. abundance was detected in N2 group of infants no matter the delivery modes or their mothers had taken antibiotics or not (Figure 5(g)).

(a, b) The bacterial α-diversity (indicated using the Shannon index) and the relative abundances of the intestinal microbial genera in infants fed by different mothers before and after the intervention. (c) The dominant microbial communities that exhibited significant differences between the groups of infants fed by different mothers were analyzed using LEfSe. The values among groups were compared by Kruskal-Wallis rank sum test. (d) The relative abundances of intestinal *Bifidobacterium* spp. in the infants of N1 and N2 groups. (e) The relative abundances of bacterial genera in infants who were exclusively or not exclusively breastfed. (f, g) Effects of maternal Abx use and the delivery mode on the α-diversity and relative abundances of intestinal microbiota in infants. All values are presented as the mean ± SD (*n* ≥ 6). The study size is indicated in the panels.

### Effects of SA+Pro intervention on HMOs and the gut microbiota in the paired mothers and infants

**Figure 6.**
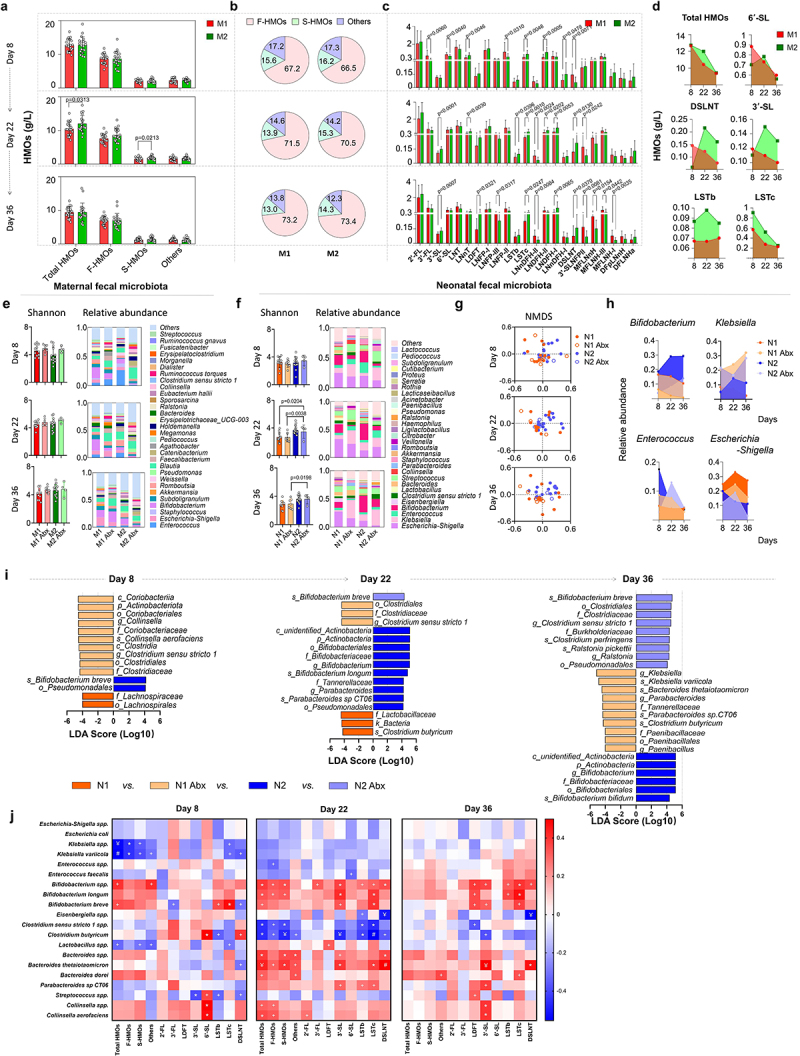
Analysis of data from the 20 paired mothers and infants.

Among all samples analyzed, some were from mothers who may have failed to follow up at certain collection points or did not exclusively breastfeed their infants. Therefore, we further analyzed the data for 20 mother – infant pairs in each group, who provided milk and fecal samples at all three time points and only breastfed their infants. The results suggested SA+Pro intake delayed the decreasing trend of HMO levels in milk from mothers in the M2 group, particularly for the S-HMOs 6′-SL, 3′-SL, DSLNT, LSTb, and LSTc (Figure 6(a–d)).

(a, b) The contents of all HMOs, F-HMOs, S-HMOs, and other HMOs and the relative abundances (%) of HMOs in milk samples from 20 mothers in each group. (c) Comparison of the major HMOs fractions in milk samples from 20 mothers in each group. Multiple comparisons were performed by using one-way ANOVA. (d) A line chart depicting the content trends of all HMOs and the major S-HMOs in milk samples from both groups. (e, f) The α-diversity (indicated using the Shannon index) and longitudinal changes in the mean relative abundances of bacterial genera in 20 mother – infant pairs. The participating infants were further divided into groups fed by mothers in the M1 and M2 groups who did or did not use Abx before giving birth or postpartum. Thus, the groups studied included the N1 and N1 Abx groups (infants), the M1 Abx and M2 Abx groups (mothers who received Abx before or after giving birth), and the N1 and N1 Abx group (infants), as displayed under the histogram. (g) The β-diversity of the intestinal microbiota in the infants was analyzed using NMDS. (h) Changing patterns in the relative abundances of the major intestinal bacterial genera in the infants of different groups. (i) The bacterial groups that exhibited significant differences among infants from different groups were analyzed using LEfSe. The values among groups were compared by Kruskal-Wallis rank sum test. (j) Correlations between maternal HMO levels and the gut microbial genera of infants before and after intervention in the mothers. ^+^*p* < .05, *p ≤ .01, ^￥^*p* ≤ .001, ^#^*p* ≤ .0001. All values are presented as the mean ± SD (*n* = 20). The study size is indicated in the panels.

The gut microbiome of the 20 mother – infant pairs in each group was also compared. SA+Pro intervention had a minor effect on the maternal intestinal microbial diversity and structures (Figure 6(e) and Supporting Information, Figure S6). However, when the intestinal microbiota of the infants was analyzed, we observed that the Shannon indices of the N2 and N2-Abx groups (whose mothers received SA+Pro intervention for 4 weeks) were substantially higher than that of the control (N1) group (Figure 6(f)). Furthermore, the abundances of conditional pathogens such as *Enterococcus* and *Escherichia-Shigella* in the N2 group infants were lower after intervention than those in the N1 group, regardless of whether their mothers had received Abx. *Bifidobacterium* spp. emerged as dominant gut bacteria in the N2 group, especially in infants whose mothers used Abx (Figure 6(f)). Non-metric multidimensional scaling (NMDS) analysis indicated that the bacterial composition of the N2 group was different from that of the N1 group and that the separation between the N1 and N2 Abx groups was significant after intervention (Figure 6(g)). The relative abundances of *Klebsiella*, *Enterococcus*, and *Escherichia-Shigella* in the infants of the N2 and N2 Abx groups decreased gradually and maintained relatively low levels (Figure 6(h)). LEfSe analysis demonstrated that *B. breve* was the most dominant species in the N2 group on day 8. However, after their mothers received SA+Pro intervention, *B. longum* (day 22) and *B. bifidum* (day 36) emerged as the most abundant *Bifidobacterium* species, which enlarged the overall dominance of the Bifidobacteriales order in the gut of these infants. Among the infants whose mothers used Abx before birth or postpartum, SA+Pro intervention also helped promote the abundance of *B. breve* and Clostridiales in the gut of the infants belonging to the N2 Abx group (Figure 6(i)).

We determined the Spearman correlation coefficient between the HMO levels in the mothers and the abundances of the top 10 microbial genera in the gut of the paired infants. On day 8, the levels of total HMOs and the levels of some S-HMOs (such as LSTb and LSTc) in the mothers’ milk correlated positively with the abundance of *Bifidobacterium* and negatively with the abundance of *Klebsiella* in the infants’ guts (Figure 6(j)). After 2 weeks of intervention, we observed that the levels of total S-HMOs, 3′-SL, LSTc, and DSLNT correlated positively with the abundances of *Bifidobacterium* and *Bacteroides* spp. and negatively with the abundances of *Clostridium*, *Escherichia-Shigella*, *Klebsiella*, and *Enterococcus* spp. These correlations were not as significant on day 36 as they were on day 22, which may be attributed to the significant decrease in HMO levels; however, high positive correlations were also observed between the 3′-SL, LSTc, and DSLNT levels and the abundance of *Bifidobacterium*.

## Discussion

In this study, we found that providing SA+Pro to lactating mothers increased S-MOs production in both maternal rats and lactating Chinese mothers through an oral-entero-mammary gland pathway, which in turn promoted the dominant growth of beneficial microbiota and the immune responses in their breastfed neonates.

In the animal study, treating lactating WT and St6gal1^±^ rats with SA+Pro increased their milk S-MO contents, which was attributed mainly to the substantial increase in 6′-SL (the major S-MO detected in rat milk). In contrast, SA supplementation alone did not increase the level of 6′-SL, Our results thus suggested that the combined intervention with probiotics may have been indispensable for converting free SAs into S-MOs, as one recent human study by Tran et al.^27^ showed that orally administered SA rapidly reaches the circulation and is excreted with the urine within hours., Higher *St6gal1* mRNA expression levels were detected in the mammary glands of maternal rats that received SA+Pro supplementation; therefore, we hypothesized that the increase production of 6′-SL was related to the regulatory effects of probiotics through the entero-mammary axis, considering that the maternal gut plays key regulatory roles in equipping the mammary glands to face the nutritional, microbiological, and immunological requirements of the growing infant.^28^ Concordantly, we observed that the levels of SCFAs (especially butyrate) and their producers such as *Lactobacillus*, *Romboutsia*, *Turicibacter*, and members of the *Peptostreptococcaceae* family ^29–33^ were higher in the gut of maternal rats that received SA+Pro intervention instead of SA supplementation alone (Figure 2).

Shively et al. demonstrated that SCFAs produced by the gut microbiota of the mother could be absorbed and transferred to the mammary glands via the gut – breast pathway.^34^ This suggests that the intake of micronutrients and macronutrients influenced the composition of bacteria residing in the gastrointestinal tract of pregnant women and that bacterial metabolites may reach the mammary glands via the entero-mammary route. Considering the regulatory effects of SCFAs on mammary epithelial cells mediated through GPCR-mediated pathways,^35^ studies on the regulatory effects of butyrate on HMO biosynthesis are crucial. Therefore, we further conducted RNA-seq analysis of the rat mammary glands from the different experimental groups and demonstrated that genes involved in medium/long-chain fatty acid biosynthesis and transportation (such as those encoding proteins in the PI3K-Akt and PPAR signaling pathways) were closely related to upregulated *St6gal1* expression and 6′-SL production following SA+Pro treatment. The results of our *in vitro* experiments with MCF-10A cells indicated that butyrate exerted effects (similar to that of SA+Pro treatment) in promoting the expression or phosphorylation of proteins related to the PI3K-Akt and PPAR signaling pathways, highlighting its indispensable role in regulating 6′-SL synthesis.

Over the past decade, the transcriptional and post-transcriptional regulation of genes involved in milk fat, protein, and lactose biosynthesis has attracted substantial research attention; however, the genes and networks that regulate milk oligosaccharides production are poorly understood. Bionaz et al. reported that a complex and interactive transcription factor network controls the expression of SREBP1-related genes in milk as the central hub.^36^ The transcription factor PPARγ is critical for regulating lipid and glucose metabolism in mammary glands, and fatty acids can activate PPARγ by binding to its ligand-binding pocket.^37^ Our results suggested that increased activation of the PI3K-AKT-mTOR pathway promoted the nuclear accumulation of SREBP1, thereby increasing medium/long-chain fatty acid synthesis in rat mammary gland promoted by SA+Pro intervention, which in turn may have activated PPARγ expression. These findings indicated that an interactive network exists between the SREBP1 and PPARγ pathways linked through medium/long-chain fatty acids. Furthermore, activation of the PI3K-Akt-mTOR signaling pathway can be regulated by GPR41, the major butyrate receptor in mammal epithelial cells.^38^ Our data from MCF-10A cells demonstrated the effects of SA+Pro treatment, as well as butyrate treatment, on the Gpr41-PI3K-Akt-mTOR-SREBP1-PPARγ signaling network. However, whether this network can regulate S-MO biosynthesis in mammary glands remains unclear. Chen et al.^39^ presented data suggesting that, in human hepatocarcinoma cells, the PI3K/AKT/mTOR signaling pathway can facilitate the formation of the RXRα–NR4A2 complex, which binds to the ST6Gal-1 promoter region to induce its transcription. We hypothesized that the well-studied heterodimeric PPARγ–RXRα complex^40^ might also interact with the ST6Gal-1 promoter region. Our ChIP analysis results conclusively suggested that PPARγ played a central role in regulating 6′-SL biosynthesis.

Mothers transfer glycans in their milk to infants through breast feeding, which helps shape the gut-microbial structure during early life. Concordantly, intervention with SA or SA+Pro significantly affected the gut microbiota and S-MO production in maternal rats, consequently leading to gut-microbe alterations in neonatal rats. *L. reuteri* was identified as the major *Lactobacillus* species that was reduced in neonates fed by St6gal1^−/−^ and St6gal1^±^ maternal rats, but it was significantly elevated in the gut of neonates fed by maternal rats (both WT and St6gal1^±^) who received SA+Pro interventions. Thongaram et al.^41^ demonstrated using *in vitro* data that *L. reuteri*, as well as probiotics supplied to maternal rats (including *L. fermentum* CECT5716, *L. rhamnoides* HN001, and *B. breve* M16 V), could not efficiently utilize HMOs such as 6′-SL. However, the cooperation of intestinal symbiotic bacteria regulated by maternal milk components may help explain its dominant growth. Our RNA-seq data suggested that *L. reuteri* can potentially modulate multiple responses of splenic T lymphocytes, suggesting a correlation exists between *L. reuteri* and elevated immune responses in neonatal rats. Previous findings confirmed that *L. reuteri* could increase the levels of butyrate, which is a vital mediator between the intestinal microbiota and host immunity,^42,43^ especially in terms of promoting the differentiation of inducible Treg cells and subsequent regulation of immune homeostasis through the butyrate-BCoA-CPT1A axis.^44^ These findings further suggested the pivotal role of *L. reuteri* in regulating the development of immune tolerance in neonatal rats.

The results of our randomized, double-blind, placebo-controlled study conducted in Northern China indicated that SA+Pro intervention led to higher s-HMO levels (i.e., 3′-SL, 6′-SL, DSLNT, LSTb, and LSTc) in the breast milk of lactating mothers than the placebo intervention. We previously reported the impact of maternal secretor status^45^ and a *Fut8* polymorphism^46^ on HMO production or N-glycan glycosylation in the milk of Chinese mothers, indicating that the human milk glycobiome is largely genetically determined. Therefore, in this study, we further evaluated the effects of SA+Pro intervention on HMO levels in secretor and non-secretor mothers. The results confirmed the stimulatory effects of SA+Pro, although, in non-secretory mothers, the increase in S-HMO levels after SA+Pro intervention was not as significant as that in secretory mothers. In 2019, Seppo et al.^19^ reported that the concentrations of 3′-FL and 3′-SL were significantly higher in the colostrum of mothers who had received probiotic supplementation during late pregnancy than those who did not. Our findings thus further verified the regulatory potential of probiotics on HMO synthesis during lactation.

Our results also suggested that the intake of SA+Pro by lactating mothers are beneficial for their newborns through promoting HMOs. *In vitro* data presented by Bode et al.^47^ suggested that 3′-SL, as an anti-inflammatory ingredient, may help to reduce the incidence of inflammatory diseases in breastfed infants. Masi et al. recently published a cohort study^48^ and reported that the feces of premature infants with higher breast milk-DSLNT levels also had higher relative abundances of beneficial bacteria (such as *Bifidobacterium*) and lower relative abundances of harmful bacteria (represented by *Enterobacter*), which is consistent with the results of this study. *Bifidobacterium* was the first probiotic bacterial community to colonize the human intestinal tract. *Bifidobacterium* spp. colonize the infant’s gut rapidly during the first few weeks of life, plays pivotal roles in regulating and maintaining homeostasis in the intestinal tract of infants, and can prevent infant allergies and inflammatory diseases.^49^ *Bifidobacterium* spp. also play vital roles in the development of intestinal microbiota and the subsequent physiological state and infant health.^50^ Laursen et al. revealed that breastmilk-promoted *Bifidobacterium* species produce aromatic lactic acids in the gut of infants and suggest that these microbial metabolites may impact immune function in early life.^51^ In this study, we observed that the abundances of *Bifidobacterium* spp. in the N2 group infants were significantly higher on days 22 and 36. *B. breve*, *B. longum*, and *Bifidobacterium infantile* are the most abundant species in the infant’s intestinal tract.^52^ Our LEfSe analysis of 20 people in each group revealed that *B. breve* had initially colonized the infant intestines by day 8. By day 22, *B. longum* had become the dominant intestinal microbe, whereas *B. bifidum* was the most abundant on day 36. Therefore, we deduced that the *Bifidobacterium* strains were transferred from the mothers’ breast milk to the infants and colonized in their intestines.^53^ However, the increased S-HMO levels in the mothers’ milk unequivocally contributed to their dominant growth. Furthermore, we observed that maternal use of Abx (prenatal or postpartum) strongly affected the gut microbiota of their infants. The abundance of *Klebsiella* in infants whose mothers had used Abx was much higher than that in the non-Abx groups, which is consistent with the published reports suggesting that the use of Abx by mothers could lead to a higher abundance of opportunistic pathogens such as *Enterococcus*, *Enterobacter*, and *Klebsiella*.^54^ However, in the gut of the N2 Abx group of infants, we observed beneficial effects on the abundance of *Bifidobacterium* spp. after maternal supplementation with SA+Pro. Similar to the situation in mothers who received SA+Pro intervention, the abundances of *Enterococcus* and *Escherichia-Shigella* were also significantly lower in the gut of their infants. Collectively, these results suggest that SA+Pro intervention strongly promoted the gut microecology of infants during lactation.

Limitations of this study: (1) Diet is one of the main drivers of gut microbiota modulation. Although in the animal study, the food provided to the maternal rats was the same, but the daily food intake of the maternal rats was not recorded, in addition, no information on diet of the human mothers was provided, which provides direction for more accurate research. (2) The translocation of probiotic strains into the rat mammary gland or the breast milk of human mothers post intervention was not detected in our study, which may be due to the imprecision of third-generation sequencing technology. In the future, the direct effects and mechanisms of probiotics on mammary epithelial cells and lactation through the gut-breast axis still need to be further explored. (3) Instead of the relative abundance of gut microbial groups, the actual microbial population and bacterial metabolome were not detected in this study. and it is suggested to keep open the possibility that other bacterial metabolites, besides SCFAs, could be involved in the regulation of HMOs biosynthesis through gut-breast axis. (4) Due to the sample size for conducting this trial was small, further studies involving large-scale cohorts to a diverse population should be conducted to confirm these effects and elucidate the underlying mechanisms.

## Conclusions

Overall, the result of our animal study, cell line experiments, and the human-cohort study confirmed that intervention with SA combined with probiotics in mothers during lactation was beneficial for both the mothers and their infants. SA+Pro intervention can improve S-HMO biosynthesis in mothers’ milk via the gut-breast axis by modulating intestinal microbial homeostasis and SCFA production as well as promote positive cooperation between multiple bifidobacterial strains during S-HMO utilization in the gut of their infants, which consequently may help enhance neonatal immune responses. Collectively, our findings may help alter the routine supplementation -practices of lactating mothers to modulate milk HMOs and promote the development of early-life gut microbiota and immunity.

## Material and methods

### Animal study

Sprague – Dawley rats (aged 8–10 weeks) were purchased from the specific-pathogen-free (SPF) animal laboratory of Dalian Medical University in Dalian, China. St6gal1^−/−^ (KO) rats were generated with a Sprague – Dawley background using clustered regularly interspaced short palindromic repeats (CRISPR)/CRISPR-associated protein 9 technology (Figure S2). They were maintained under SPF conditions that ensured lighting for 12 h/day, adequate water and food supply, and a room temperature controlled at approximately 24 ± 1°C. The food supply to rats was the Lab Mice Growth and Breeding Diet (SFS9112, Jiangsu Xietong Pharmaceutical Bio-engineering Co., Ltd., Nanjing, China). The main ingredients are corn, wheat, imported fish meal, chicken meal, soybean oil, amino acids, vitamins and minerals etc. The protein, fat, and carbohydrate energy ratios of growth feed were 22.8%, 13.8%, and 63.4% per kilogram, and total calories were 3,656 Kcal/kg. All experiments were performed using protocols approved by the Committee for Animal Care and Use at Dalian Medical University (approval number SYXK [Liao] 2018–0002) and in accordance with the 1996 National Institutes of Health Guide for the Care and Use of Laboratory Animals.

After giving birth, each maternal rat and its neonates were housed in the same cage to ensure lactation for 2 weeks. In week 3, different interventions were applied to the maternal rats in various experimental groups. The maternal rats in the CON group were gavaged with 2 ml of saline alone, the SA (sialic acid) group of maternal rats was gavaged with 2 ml of sialic acid (33.325 mg·kg^−1^·day^−1^ in saline), and the SA+Pro group was gavaged with 1 ml sialic acid (33.325 mg·kg^−1^·day^−1^ in saline) and 1 ml probiotics in saline. The probiotics contained a combination of *L. fermentum* CECT5716, *L. rhamnoides* HN001, and *B. breve* M16V at a proportion of 1:9:5, total 3 × 10^9^ colony-forming units (CFUs). The SA we used in this study was sodium sialic acid form of Neu5Ac with a purity of > 98% that was obtained commercially from BYHEALTH Co., Ltd. China. It was produced from food grade glucose and corn syrup through fermentation (by *Escherichia coli* SA-8), filtration, sterilization, hydrolysis, purification, and other processes. All groups received 2 weeks of continuous intervention. The maternal rats and their neonates (N) were euthanized on day 28 of lactation (4 weeks) by injection of pentobarbital sodium.

### Sample collection

Rat blood samples were obtained from the medial canthus vein. Serum samples were obtained by centrifuging the blood samples at 3,000 rotations/min (rpm) for 15 min and subsequently stored at − 80°C refrigerator (Thermo Scientific™, USA) before analysis. Rats were anesthetized and bled out through the abdominal aorta. The spleens were immediately obtained for flow cytometric analysis. Mammary glands and rat milk samples were immediately frozen at − 80°C. The total colonic contents of each rat were collected and preserved at − 80°C.

### Randomized, double-blind, placebo-controlled supplementation trial

#### Participants and study design

The characteristics of the eligible participants and their infants enrolled in this randomized, double-blind, placebo-controlled trial are presented in Table 1. Informed consent was obtained from each mother. Ethical approval was obtained from the Research Ethics Committee of Dalian Women and Children Medical Center (Group) (Dalian, Liaoning Province; approval number 2,020,018).

#### Randomization, blinding, and study products

For this trial, eligible mothers were randomly allocated (1: 1) to the placebo group (M1) and the intervention group (M2). The randomization sequences were generated computationally by the principal investigator of the study and were not accessible to other investigators of the study. Participants and staff were kept unaware of these sequences until the completion of data analysis.

The intervention began on day 8 of lactation (baseline) and was provided continuously for 4 weeks. During the trial, three visits were conducted on the 8th day (before intervention), the 22nd day (intervention for 2 weeks), and the 36th day (intervention for 4 weeks) of lactation. Breast milk, maternal feces, infant feces, and infant saliva samples were collected, transported immediately in ice boxes to Dalian Medical University, and stored at − 80°C until analysis according to our previous study.^37^ Clinical examinations were conducted, and anthropometric data were obtained on the last visit. The M1 group of mothers were provided 1.5 g (one package/day) of placebo powder containing maltodextrin (420 mg), resistant dextrin (810 mg), and polydextrose (270 mg). The M2 group of mothers were provided 1.5 g (one package/day) of SA+Pro powder containing SA (in sodium salt form, 300 mg, 50% purity, 50% maltodextrin) and probiotics (*L. fermentum* CECT5716, *L. rhamnosus* HN001, and *B. breve M*-16 V at relative proportions of 1:9:5, 1.5 × 10^10^ CFUs) based on resistant dextrin (810 mg) and polydextrose (270 mg) powder. The SA+Pro product (a patented combination of SA and 3 probiotic strains, with a trademark name of “PtSyne”) was obtained commercially (BYHEALTH Co., Ltd). The product license number is SC-12744040400094, and the Executive Standard number is Q/TCBJ0046S. The final preparations of SA+Pro and placebo products were provided to participants in foil-sealed commodity packages with the same appearance. They were only labeled with random numbers and did not provide information related to the trial. All products were stored at 4–8°C refrigerator (Haier, Shandong, China) before distribution, and participants were instructed to refrigerate them throughout the 4-week study.

### Detecting milk oligosaccharides

The entire mammary glands of maternal rats were gently peeled off after sacrificing the animals, incubated in 5 ml triple-distilled water at 45°C, and placed in a water bath at 45°C for 5 min. Subsequently, the milk samples were homogenized using an ultrasonic cell grinder (BiLon, Shanghai, China) until the rat milk was extracted completely and stored immediately at − 80°C.

To detect oligosaccharides, extracted rat and human milk samples (approximately 2 ml) were first centrifuged at 8,000 rpm for 10 min at 4°C. After removing the top lipid layer, the defatted milk solution was freeze-dried. Subsequently, 1 ml ethanol/water (2:1) was added to the dried powder, and the mixture was centrifuged at 8,000 rpm for 10 min at 4°C to remove most of the proteins. The supernatant was dried and redissolved in 50 μl 50% acetonitrile (ACN)/H_2_O solution. The resulting mixture was centrifuged, and the supernatant was used for further analysis.^17^ A solid-phase extraction system with hydrophilic interaction chromatography and a collision-induced dissociation tandem electrospray tandem-mass spectrometry system were used to detect the S-MOs in the rat milk samples.^55^ The HMOs were detected via online liquid chromatography-mass spectrometry, as described previously.^56,57^

### Cell culture and exposure to different substances

The human MCF-10A mammary epithelial cell line (American Type Culture Collection, CRL-10317) was grown in Dulbecco’s modified Eagle’s medium (Gibco, Thermo Fisher Scientific, USA) supplemented with 10% fetal bovine serum (BioInd, Israel) and 1% penicillin-streptomycin (Procell, Procell Life Science&Technology Co., Ltd., Wuhan, China). The cell line was maintained at 37°C in a humidified atmosphere containing 5% CO_2_ and 95% air. The cells were plated into 6-well cell culture plates at a density of 10^5^ cells/cm^2^, 1 × 10^6^ cells·ml^−1^ and incubated in a 5% CO_2_ incubator at 37°C for 24 h, after which they were incubated with different concentrations of substances for 24 h (or without added compounds in control experiments). The final concentrations of But were 0.5, 0.05, and 0.005 μmol·L^−1^, and those of SA were 50, 5, and 0.5 μg·ml^−1^. SA was combined with the probiotics *L. fermentum* CECT5716, *L. rhamnosus* HN001, and *B. breve M*-16 V (at a 1:9:5 ratio) at final concentrations of 0.1, 0.01, or 0.001 mg·ml^−1^ (for SA) or 0.33 × 10^6^, 3 × 10^6^, 1.67 × 10^6^, 0.33 × 10^5^, 3 × 10^5^, 1.67 × 10^5^, 0.33 × 10^4^, 3 × 10^5^, or 1.67 × 10^4^ CFUs·ml^−1^ (for the probiotics). All substances were filtered through 0.22-μm polyethersulfone membrane syringe filter (Millex-GP, Germany). For PPARγ and PI3K pathway inhibition, cells that reached approximately 70% confluency were incubated with butyric acid and SA+Pro (same concentrations as described earlier) for 15 min, after which PPARγ and PI3K-Akt inhibitors (T0070907 and LY294002, respectively, Abmole, Shanghai, China) were added, and total RNA was extracted 24 h later.

### Reverse transcription-quantitative polymerase chain reaction (RT-qPCR)-based detection of the glycosyltransferase gene

Total RNA was extracted from the mammary glands tissue of the maternal rats using Trizol (TransGen, TransGen Biotech Co., Ltd., Beijing, China). Complementary DNA was generated using the cDNA synthesis SuperMix Kit (TransGen, TransGen Biotech Co., Ltd, Beijing, China) following the manufacturer’s instructions. qPCR was conducted using PerfectStart^TM^ Green qPCR SuperMix (TransGen, TransGen Biotech Co., Ltd, Beijing, China) in a LightCycler96 Real-Time PCR machine (Roche, Switzerland). The results were calculated using the 2^−ΔΔCt^ method. The sequences of the primers (5′–3′) used in this study were listed in Table 3. The primers were synthesized by Sangon Biotech (Shanghai, China).

**Table 3.** Primers used for q-PCR detection of glycosyltransferase genes expressed in mammary epithelial cells.

Gene	Primer sequence	Tm	Product	Reference
Stsgal1 (Rat)	Forward: GTACCCTGCTGTCCTTCCTGTTTC Reverse: CTCTCCACTGCCCTCCCTTGTAG	63 °C	91 kda	21
St3gal1 (Rat)	Forward: CTGACAGTCCACAACGCTCT Reverse: AGGCCCATACGAGGAGTCTT	60 °C	219 kda	22
B4galt1 (Rat)	Forward: AACGTTGGCTTTCAAGAGGC Reverse: ACGTAAGGCAGGCTAAACCC	59 °C	164 kda	This study
Lgals3 (Rat)	Forward: TATCCTGCTACTGGCCCCTT Reverse: CCAATGATCCCCAGTTGGCT	60 °C	413 kda	This study
Gcnt1 (Rat)	Forward: CACAATCTGGCTTCTGCTTTCT Reverse: GATGTGTTGACCACCGGCTA	60 °C	260 kda	This study
β-actin (Rat)	Forward: ATCCTCTTTGTCTTTACGGTT Reverse: GGGGTGTGAAGGTCTCAAACATG	61 °C	413 kda	This study
Fut2 (Human)	Forward: ATCCTCTTTGTCTTTACGGTT Reverse: TCCCAGTGCCTTTGATGTTG	56 °C	123 kda	23
Fut4 (Human)	Forward: TGGCATGTAGGAAGCACCTG Reverse: GCACGTGGAACTAGGAGGTC	60 °C	230 kda	24
St6gal1 (Human)	Forward: TTTTTGCCTTTGCAGATGAGTT Reverse: TCAGACCCCATGGCCAATTT	58 °C	373 kda	25
St3gal1 (Human)	Forward: TTCCCTCCCCGCTATACCAA Reverse: TTCGTGGCATGTTGGCTTTG	60 °C	225 kda	This study
B4galt4 (Human)	Forward: ACTTCGTGGGTGCCATTCAAGAGA Reverse: AAGGAGACACAGAAGGGCAGTTGT	64 °C	150 kda	This study
B3gnt2 (Human)	Forward: ACTTCGTGGGTGCCATTCAAGAGA Reverse: AAGGAGACACAGAAGGGCAGTTGT	57 °C	112 kda	26
Gadph (Human)	Forward: TGCTGGGGAGTCCCTGCCACA Reverse: GGTACATGACAAGGTGCGGCTC	65 °C	119 kda	This study

### Western blot analysis

Protein concentrations were determined using a BCA Kit (Beyotime, Beyotime Biotechnology, Shanghai, China). Equal amounts of proteins were subjected to sodium dodecyl sulfate-polyacrylamide gel electrophoresis and transferred to polyvinylidene fluoride membranes. Antibodies against GPR41 (66811–1-Ig, Sanying, WuHan, China) PI3K (A4992, ABclonal, Wuhan, China), phosphorylated (p)-PI3K (Ab278545, Abcam, UK), Akt (A18675, ABclonal, Wuhan, China), p-Akt (AP1208, ABclonal, Wuhan, China), mTOR (A11355, ABclonal, Wuhan, China), p-mTOR (AP0115, ABclonal, Wuhan, China), RxRα (A19105, ABclonal, Wuhan, China), SREBP1 (A5754, ABclonal, Wuhan, China), PPARγ (A16958, ABclonal, Wuhan, China), St6gal1 (A5754, ABclonal, Wuhan, China), and GAPDH (AB-P-R001, Xianzhi, Hangzhou, China) were used as primary antibodies. All primary antibodies were used at a 1:1000 dilution except for those against p-PI3K (1:500 dilution) and GPR41 (1:2000 dilution). Goat anti-rabbit-HRP (BA1054, Boster Bio, Wuhan, China) and goat anti-mouse-HRP (BA1051, Boster Bio, Wuhan, China) were used as secondary antibodies. The bands were visualized using an Enhanced Chemiluminescence (ECL) Kit (Applygen, Applygen Technologies Co., Ltd., Beijing, China). The protein bands were densitometrically analyzed using the IPP software.

### ChIP assays

ChIP assays were performed using the ChIP Assay Kit (Beyotime, Beyotime Biotechnology, Shanghai, China) following the standard protocol. Protein – DNA complexes were incubated with 5 μg of anti-PPARγ antibody, anti-RXRα antibody, or normal rabbit IgG (provided in Beyotime ChIP Assay Kit) as the immunoprecipitating antibodies. Purified DNA was subjected to PCR analysis using the following ChIP primer sequences: 5′-AGGCATTTAGGGGTCCTTGC-3′ and 5′-AGAGTTGCGAAGACTGGGTC-3′.^39^ The amplified products were separated by 2% agarose gel electrophoresis and analyzed using Image Lab software.

### ELISA analysis

IL-6, IL-8, IL-10, interferon-gamma (IFN-γ), transforming growth factor β (TGF-β), sIgA, and lactoferrin (LF) levels in rat serum and milk, and saliva sIgA levels of infants were measured using ELISA kits (Jiangsu Meibiao Biotechnology Co., Ltd, Jiangsu, China) according to manufacturer’s instructions. The absorbance was measured at 450 nm using an Enzyme labeling instrument (Thermo Scientific™, USA).

### Flow cytometry-based detection of lymphocyte subsets of neonatal rats

Fresh spleens and MLNs from neonatal rats were ground into single-cell suspensions in phosphate-buffered saline (PBS). Subsequently, the cells were collected and centrifuged at 2,500 rpm for 5 min. The supernatants were discarded, and red blood cell lysis buffer (Solarbio, Solarbio Technology Co., Ltd., Beijing, China) was added to remove red blood cells, after which the remaining cells were washed twice with PBS. Subsequently, the cells (1 × 10^6^ were analyzed by flow cytometry. Briefly, the cells were incubated with 3% bovine serum albumin (Solarbio, Solarbio Technology Co., Ltd. Beijing, China) for 40 min to block Fcγ receptors. Subsequently, the cell suspensions were incubated with different antibodies, including APC-anti-rat CD45R, FITC-anti-rat CD3, FITC-anti-rat CD4, or APC-anti-rat CD8 (1:100 dilution for each antibody, eBiosciences, USA) on ice for 40 min without light. Flow cytometry was performed using the FACS Calibur C6 Plus instrument (Becton Dickinson, Mountain View, CA, USA), and the data were analyzed using FlowJo software (Tree Star).

### Immunizing neonatal rats

Following 4 weeks of lactation, the 5-week-old neonatal rats were immunized by subcutaneous injection with 200 μg of OVA (Sigma-Aldrich, Germany) in 200 μl PBS mixed with an equal volume of Freund’s complete adjuvant (Sigma-Aldrich, Germany). Two weeks later, these rats were immunized again with 200 μg OVA in 200 μl PBS, again by intraperitoneal injection. Serum samples were collected 28 days after immunization.

### Fecal DNA extraction, PCR, and 16S rRNA gene sequencing and analysis

Total microbial genomic DNA was extracted from rat colonic contents and feces from mothers and newborns using the E.Z.N.A.® Stool DNA Kit (Omega Bio-Tek, USA), QIAamp DNA Stool Mini Kit (Qiagen GmbH, Germany) was used for extraction of total DNA from rat milk, following the manufacturer’s instructions. After the quality of the isolated DNA samples was examined, they were used as templates for amplifying the gut bacterial V3–V4 region with the following PCR primers: 515 F: 5′-GTGYCAGCMGCCGCGGTAA-3′ and 806 R: 5′-GGACTACNVGGGTWTCTAA-3′. PCR amplicon sequences were analyzed using the Illumina NovaSeq platform (Novogene Bioinformatics Technology Co., Ltd., Tianjin, China), and PCR amplicon sequences derived from rat milk were sequenced using the Illumina HiSeq platform (Personal Biotechnology Co., Ltd., Shanghai, China). The sequences obtained after quality-control analysis was studied further using Quantitative Insights Into Microbial Ecology software (version 1.9.1). Subsequently, operational taxonomic unit (OTU)-clustering and species-classification analysis were performed. Based on the OTU-clustering results, species were annotated using a representative sequence for each OTU, and the community composition of each sample was assessed at each classification level. The OTUs of representative sequences at a similarity cut off of 97% and their relative abundance (alpha-diversity) were used to calculate Shannon and other indexes by UCLUST. The abundance and diversity of the OTUs (beta-diversity) were examined using Principal coordinates analysis (PCoA) with unweighted UniFrac analysis or Non-metric multidimensional scaling (NMDS) analysis in the R software. The statistical significance of the separation among groups was assessed by the linear discriminant analysis effect size (LEfSe) method based on linear discriminant analysis scores exploited, which used the nonparametric factorial Kruskal – Wallis and Wilcoxon rank sum test to identify key OTUs for separating different treatment groups at a significance level of 0.05. Correlations between the gut microbiota of infants and the maternal milk oligosaccharides were tested using Spearman rank correlation and visualized using a heatmap. The correlation coefficient (r) indicates the degree of correlation between two variables. The higher the absolute value of the *r* value means the higher the correlation. *p* value < .05 was considered as a significant correlation.

### Measuring fecal SCFA levels

The colonic SCFA contents of rats in the different experimental groups were detected using gas chromatography-mass spectrometry (GC-MS). Standard curves were plotted using different concentrations of Ace, propionic acid, isobutyric acid, But, isovaleric acid, and valeric acid as standards. The samples were mixed with 2 ml of solution (1:3, water: phosphoric acid), homogenized by vortexing for 2 min, extracted with 1 ml of ether for 10 min, and centrifuged for 20 min. Subsequently, 1 ml ether was added for extraction, and the samples were centrifuged for 10 min at 4000 rpm/min. Both extraction solutions were combined and volatilized to a volume of 1 ml, injected into the GC-MS instrument, and analyzed. SCFAs were analyzed using a Thermo Fischer GC-MS ISQ LT instrument equipped with a TG WAX column. The temperature program was as follows: 100°C (5 min), followed by a temperature increase of 5 °C/min to 150°C (hold time: 0 min) and a further increase at 30 °C/min up to 240°C (hold time: 30 min). The ion-source temperature was 200°C and the transfer-line temperature was 250°C. The carrier-gas flow rate was 1 ml/min, and the shunt ratio was 75:1.

### Culturing an L. reuteri strain in vitro

The standard *L. reuteri* strain used in this study (BNCC192190) was obtained from Beina Chuanglian Biotechnology Co., Ltd. After activation in lactic acid bacteria culture MRS medium (Hopebio, Qingdao Hope Bio-Technology Co., Ltd., Shandong, China), the strain was inoculated (1%) into sugar-free MRS broth containing 0.1% l-cysteine (as a control) or sugar-free MRS (Hopebio, Qingdao Hope Bio-Technology Co., Ltd., Shandong, China) broth supplemented with 0.5 mg/mL of 6′-SL (Huicheng Technology Co., Ltd., Shanghai, China), after which it was cultured at 37°C. Optical density was measured at 620 nm to monitor the growth of the strain under different conditions.

### RNA-seq and analysis

To analyze the effect of *L. reuteri* on immune cells, total lymphocytes were isolated from the spleen of 4-week-old rats, and 2–3 × 10^6^ cells were cultured in the complete Roswell Park Memorial Institute-1640 medium (Procell, Life Science & Technology Co., Ltd., Wuhan, China). Total lymphocytes were divided into a control group and an *L. reuteri* group, and the effects of *L. reuteri* metabolites were studied via RNA-seq analysis. In the *L. reuteri* group, after filtration, the *L. reuteri* metabolites were added to the cell culture medium at a multiplicity of infection of 1:10 and tested using three biological replicates. The cells in both groups were co-cultured at 37°C in 5% CO_2_ cell incubator for 6 h and were collected and washed with PBS.

Total RNA was extracted using the RNA Nano 6000 Assay Kit, and an Agilent Bioanalyzer 2100 system was used to check the RNA integrity (Agilent Technologies, USA). Sequencing libraries were generated using the NEBNext® Ultra^TM^ RNA Library Prep Kit for Illumina® (New England Biolabs, Beijing, China) following the manufacturer’s recommendations. The clustering of different groups of samples was performed using a cBot Cluster Generation System and a HiSeq X-Ten/NovaseqS4 PE Cluster Kit (Illumina, USA) according to the manufacturer’s instructions. After cluster generation, the library preparations were sequenced using the Illumina NovaSeq 6000 platform, and 150-bp paired-end reads were generated. Differentially expressed mRNAs were identified based on a log_2_ fold-change in the expression of > 1 or a p-value of < .05, using the DESeq2 R package (1.20.0). The differentially expressed genes were subjected to GO enrichment analysis.

### Statistical analysis

All data are presented as the mean ± standard deviation (SD; *n* ≥ 3). GraphPad Prism software, version 8.1 (Graph Pad Software), was used for statistical analysis and comparing differences in the experimental data. A non-parametric t-test was used to compare the two groups, and one-way analysis of variance and Tukey’s test were used to identify statistical differences between the two groups. Spearman rank correlation analysis was employed to assess correlation between the gut bacteria of infants and the milk oligosaccharides of mothers. *p* < .05 were considered to reflect statistically significant differences.

## Abbreviations

6′-SL: 6′-sialyllactose
SD: Abx: antibiotic use
Ace: acetic acid
But: butyric acid
CB: cesarean section
CFU: colony-forming unit
ChIP: chromatin immunoprecipitation
CON: control
CRISPR: clustered regularly interspaced short palindromic repeats
DSLNT: disialyllacto-N-tetraose
ELISA: enzyme-linked immunosorbent assay
F-HMO: fucosylated HMO
Fuc: fucose
Gal: galactose
GC-MS: gas chromatography-mass spectrometry
Glc: glucose
GlcNAc: *N*-acetylglucosamine
GO: Gene Ontology
Gpr: G-protein coupled receptor
H: heterozygous
H2A1: total sialylated milk oligosaccharide
HMO: human milk oligosaccharide
IFN-γ: interferon gamma
IL: interleukin
KEGG: Kyoto Encyclopedia of Genes and Genomes
KO: knockout
*L. reuteri*: *Lactobacillus reuteri*
LEfSe: linear discriminant analysis effect size
LF: lactoferrin
LSTc: sialyllacto-*N*-neotetraose c
M1: placebo group
M2: SA+Pro intervention group
MLN: mesenteric lymph node
N1: neonatal control group
N2: neonatal SA+Pro group
NANA: *N*-acetylneuramic acid
NMDS: non-metric multidimensional scaling
Non-Abx: non-antibiotic-using
OUT: operational taxonomic unit
OVA: ovalbumin
P: phosphorylated
PBS: phosphate-buffered saline
PCoA: principal coordinate analysis
Pro: probiotic
RNA-seq: RNA-sequencing
Rpm: rotations/min
RT-Qpcr: reverse transcriptase-quantitative polymerase chain reaction
S-HMO: sialylated human milk oligosaccharide
S-MO: sialylated milk oligosaccharide
SA: sialic acid
SCFA: short-chain fatty acid
SD: standard deviation
SE-: non-secretor mother
SE+: secretor
SIgA: secretory immunoglobulin A
SPF: specific-pathogen free
TGF-β: transforming growth factor
Treg: regulatory T
VB: vaginal birth
WT: wild-type.

## Supplementary Material

Supplemental Material
