# Supplementary figures and images for: Sialic acid-based probiotic intervention in lactating mothers improves the neonatal gut microbiota and immune responses by regulating sialylated milk oligosaccharide synthesis via the gut–breast axis
Source: Gut Microbes. 2024 Apr 17;16(1):2334967. doi: 10.1080/19490976.2024.2334967 (PMC11028031; doi:10.1080/19490976.2024.2334967)

Figure S1


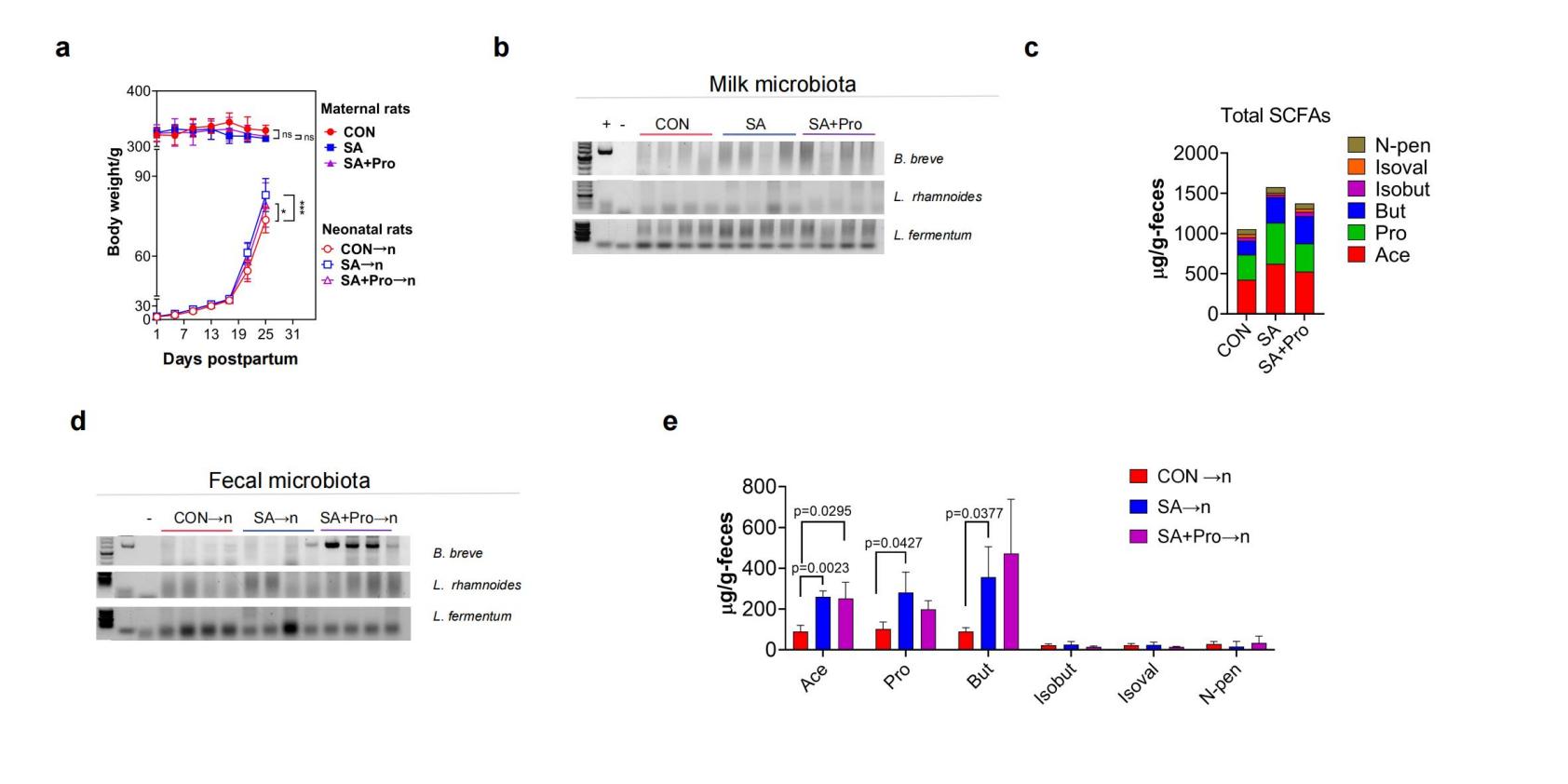


Figure S2


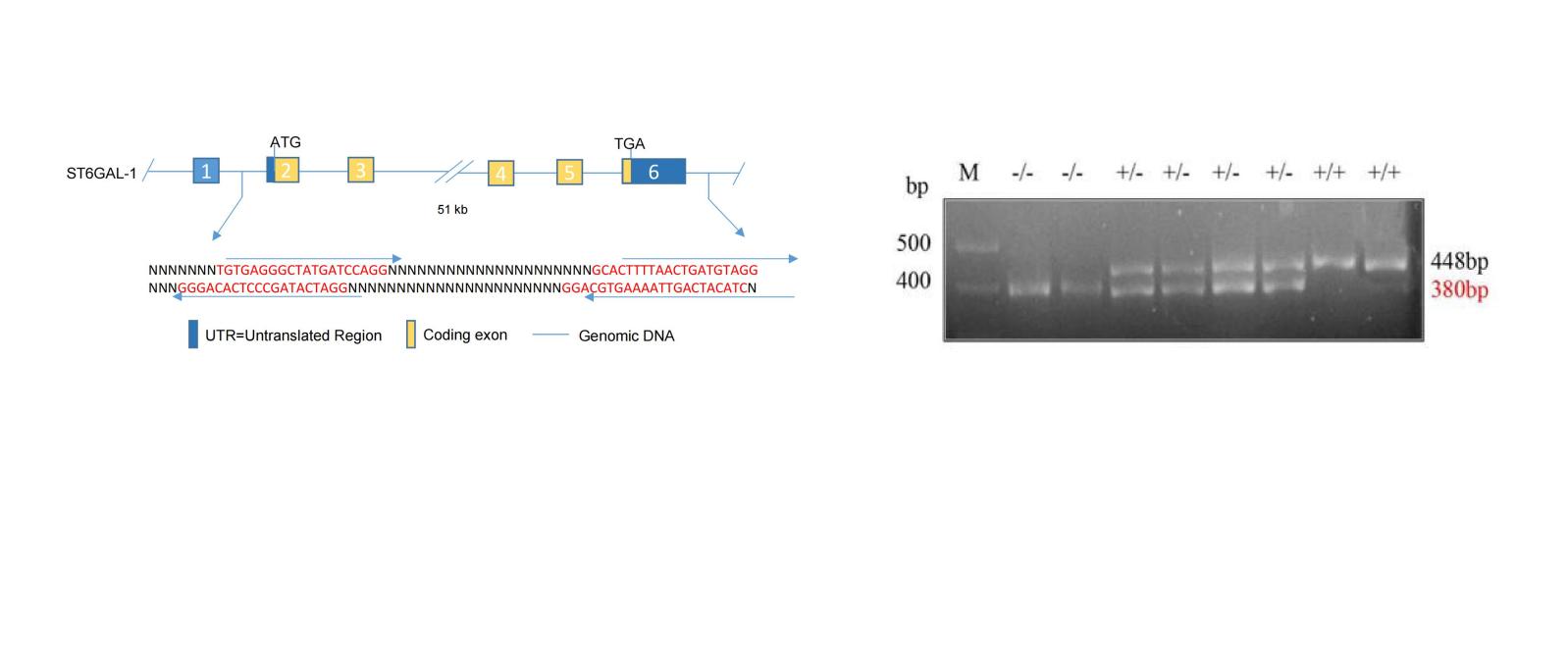


Figure S3


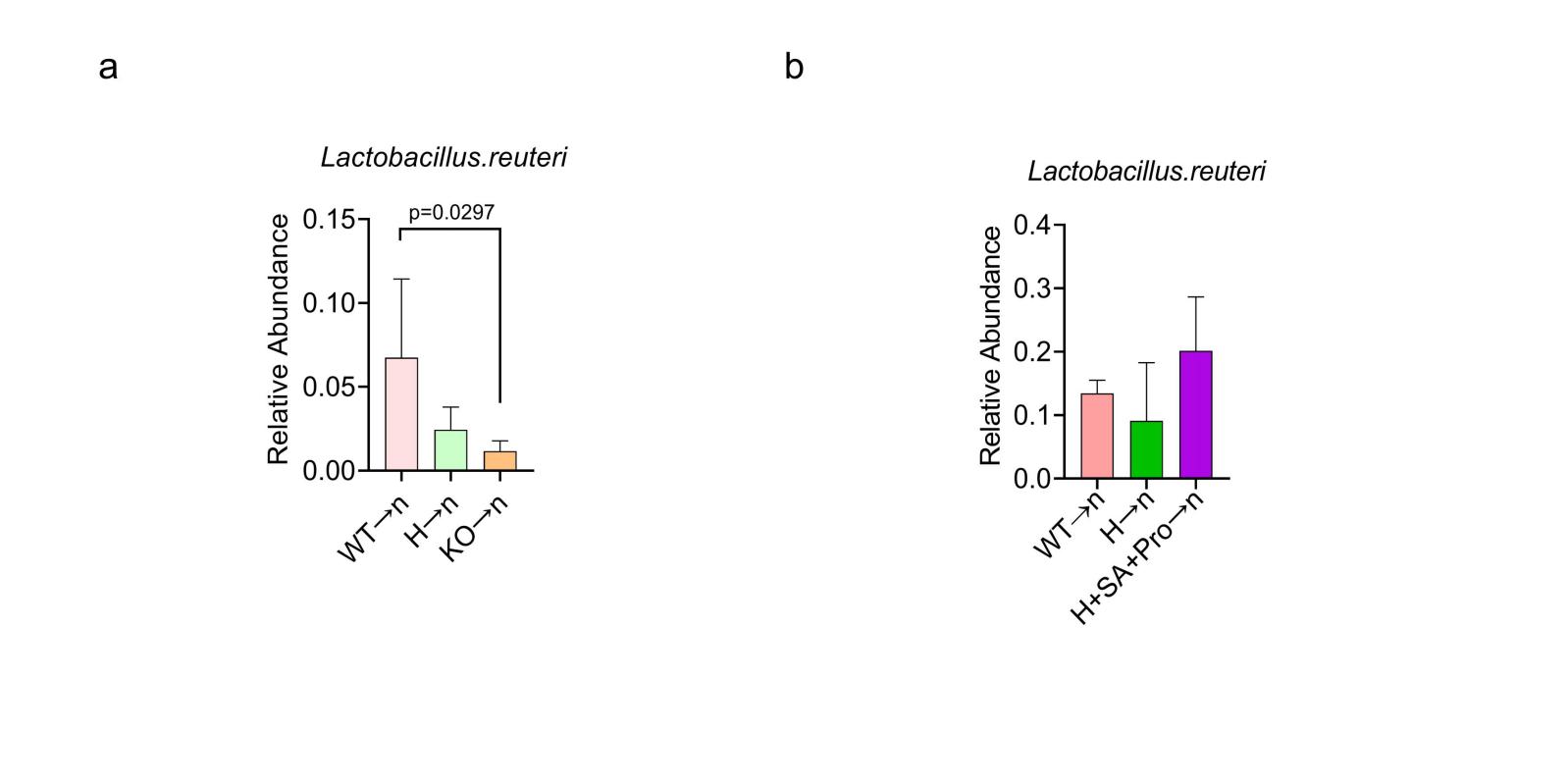


Figure S4

Figure S5


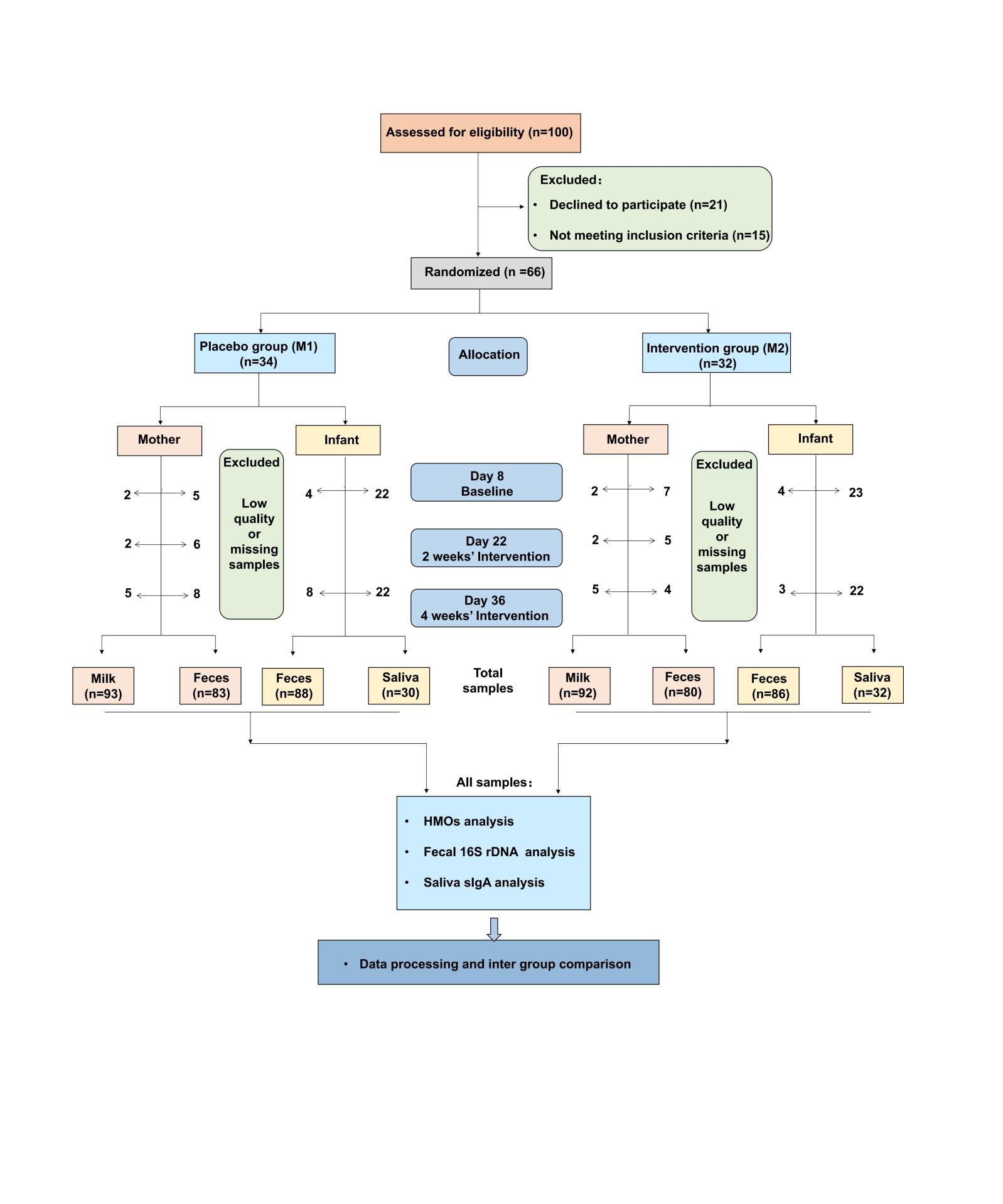


Figure S6


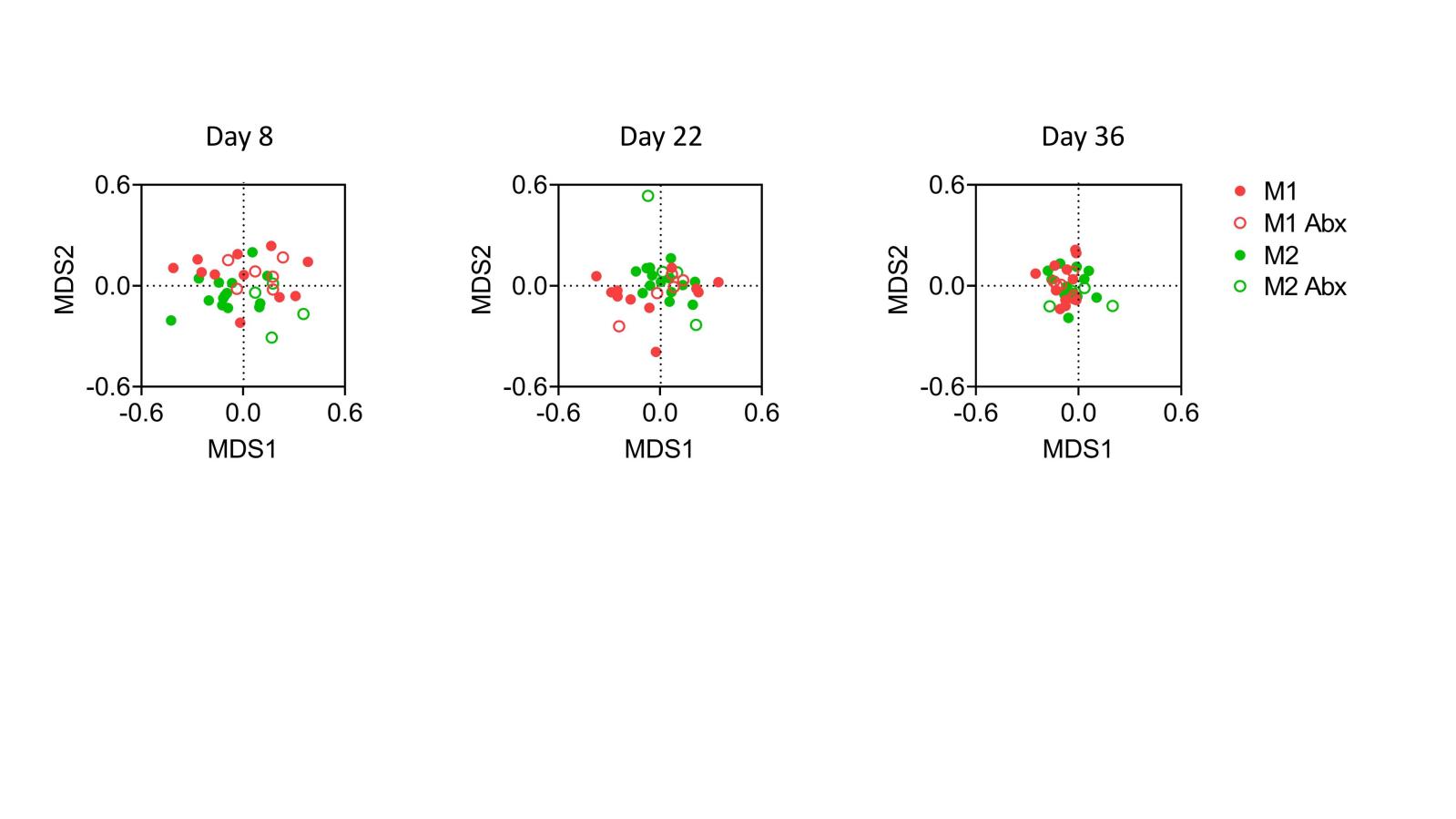

Supplement: Supplemental Material [file KGMI_A_2334967_SM7717.docx]
